# Human germ/stem cell-specific gene *TEX19* influences cancer cell proliferation and cancer prognosis

**DOI:** 10.1186/s12943-017-0653-4

**Published:** 2017-04-26

**Authors:** Vicente Planells-Palop, Ali Hazazi, Julia Feichtinger, Jana Jezkova, Gerhard Thallinger, Naif O. Alsiwiehri, Mikhlid Almutairi, Lee Parry, Jane A. Wakeman, Ramsay J. McFarlane

**Affiliations:** 1 North West Cancer Research Institute, School of Medical Sciences, Bangor University, Brambell Building, Deiniol Road, Bangor, Gwynedd LL57 2UW UK; 2 Computational Biotechnology and Bioinformatics Group, Institute of Molecular Biotechnology, Graz University of Technology, Graz, Austria; 3 Omics Center Graz, BioTechMed Graz, Graz, Austria; 4 European Cancer Stem Cell Research Institute, Cardiff University, Hadyn Ellis Building, Maindy Road, Cardiff, CF24 4HQ UK; 5 Present address: Department of Zoology, King Saud University, Al-Ryiadh, Saudi Arabia

**Keywords:** Cancer-testis gene, Cancer prognosis, Cell proliferation, Soma-to-germline transition, TEX19

## Abstract

**Background:**

Cancer/testis (CT) genes have expression normally restricted to the testis, but become activated during oncogenesis, so they have excellent potential as cancer-specific biomarkers. Evidence is starting to emerge to indicate that they also provide function(s) in the oncogenic programme. Human *TEX19* is a recently identified CT gene, but a functional role for TEX19 in cancer has not yet been defined.

**Methods:**

siRNA was used to deplete TEX19 levels in various cancer cell lines. This was extended using shRNA to deplete TEX19 in vivo. Western blotting, fluorescence activated cell sorting and immunofluorescence were used to study the effect of TEX19 depletion in cancer cells and to localize TEX19 in normal testis and cancer cells/tissues. RT-qPCR and RNA sequencing were employed to determine the changes to the transcriptome of cancer cells depleted for TEX19 and Kaplan-Meier plots were generated to explore the relationship between *TEX19* expression and prognosis for a range of cancer types.

**Results:**

Depletion of TEX19 levels in a range of cancer cell lines in vitro and in vivo restricts cellular proliferation/self-renewal/reduces tumour volume, indicating TEX19 is required for cancer cell proliferative/self-renewal potential. Analysis of cells depleted for TEX19 indicates they enter a quiescent-like state and have subtle defects in S-phase progression. TEX19 is present in both the nucleus and cytoplasm in both cancerous cells and normal testis. In cancer cells, localization switches in a context-dependent fashion. Transcriptome analysis of TEX19 depleted cells reveals altered transcript levels of a number of cancer-/proliferation-associated genes, suggesting that TEX19 could control oncogenic proliferation via a transcript/transcription regulation pathway. Finally, overall survival analysis of high verses low *TEX19* expressing tumours indicates that *TEX19* expression is linked to prognostic outcomes in different tumour types.

**Conclusions:**

TEX19 is required to drive cell proliferation in a range of cancer cell types, possibly mediated via an oncogenic transcript regulation mechanism. *TEX19* expression is linked to a poor prognosis for some cancers and collectively these findings indicate that not only can *TEX19* expression serve as a novel cancer biomarker, but may also offer a cancer-specific therapeutic target with broad spectrum potential.

**Electronic supplementary material:**

The online version of this article (doi:10.1186/s12943-017-0653-4) contains supplementary material, which is available to authorized users.

## Background

The ability of cancer cells to self-renew and undergo phenotypic changes has led to the postulate that some have similarities to germline cells and/or stem cells [1, 2], leading to the suggestion that a key feature of oncogenesis is a cellular soma-to-germline transition [1, 3–6]. This is supported by the finding that tumours in *Drosophila melanogaster* activate a large cohort of germline genes during oncogenesis and that some of these are essential for tumour progression [7–9]. Analysis of gene expression in human tumours indicates that a similar pattern of germline gene activation is also apparent, inferring a possible functional requirement [3].

One major group of germline genes is termed the cancer/testis (CT) genes. These encode cancer/testis antigens (CTAs), proteins that are normally only present in healthy adult testis, but are also found in a wide range of cancers [10–12]. Little is currently known about the function in the testis for most of these proteins, but evidence is emerging to indicate that CTAs function in oncogenic processes, supporting the idea of a functional soma-to-germline transition [12, 13]. Examples include regulation of cellular mitotic fidelity [14–20] and invasiveness [21–25]. These findings offer attractive new avenues for cancer-specific therapeutic targeting via inhibition of oncogenic CTA functions [2, 11–13].

Recently, a pipeline for the identification of new CT genes was developed [26, 27]. One of the genes identified was *T*estis *Ex*pressed *19* (*TEX19*), a mammalian specific gene with a poorly defined function [28, 29]; subsequently, this expression profile was verified [30] and TEX19 protein was shown to be a CTA [31].

In rodents, the *TEX19* orthologue has undergone duplication to generate a paralogue pair of genes, *Tex19.1* and *Tex19.2* [29]. Both murine genes are differentially expressed, with *Tex19.2* expression restricted to the developing gonadal ridge and adult testis and *Tex19.1* being expressed in the adult testis, the placenta and in the early embryo, in a pattern matching the pluripotency marker gene *Oct4* [29], although expression control mechanisms of the two genes is distinct [32]. *Tex19.1* is expressed in embryonic stem cells (ESCs) and whilst this might infer a functional role in stemness [29], *Tex19.*1^-/-^ ESCs have no overt stemness/ proliferative defects [33], nor are there any overt phenotypic defects in spermatagonial germ cells [34]. Preliminary analysis of human *TEX19* expression indicates it is orthologous to *Tex19.1* as it is expressed in human ESCs [29]. Tex19.1 is largely cytoplasmic and appears to be located in spermatagonial germline cells of testis seminiferous tubules, with levels diminishing as cells differentiate during spermatogenesis, suggesting a germline-specific function [29, 33, 34].

*Tex19.1* ^-/-^ mice are viable with no apparent behavioural defects [33–35]. There is a slight increase in mortality of pups older than 5 days post-partum, but this has been attributed to in utero developmental defects linked to placental dysfunction [33, 35]. Female fertility of *Tex19.1* ^-/-^ mice has been independently reported to be reduced [34] and normal [33], with the discrepancy being attributed to distinct genetic backgrounds [33]. Males exhibit sub-normal levels of fertility with considerable inter-individual differences in spermatogenesis indicating a phenotypic variability, the cause of which is unknown [33, 34]. Meiosis in *Tex19.1* ^-/-^ males has defects, which include impaired meiotic chromosome synapsis, the persistence of unprocessed DNA double-strand breaks, increased apoptosis and post pachytene meiosis I chromosome segregation defects, although these were not uniformly apparent [33, 34]. Analysis of gene expression during early spermatogenesis did not reveal any notable changes to genes that could directly influence meiosis, but there was significant elevation in the expression of the class II long terminal repeat (LTR)-retrotransposon *MMERVK10C* [33, 34]. Expression of other transposable elements (TEs), such as *LINE*s, *SINE*s and *IAP* retrotransposons did not appear to be altered, indicating TE specific suppressor activity for Tex19.1 in testis, which is proposed to be distinct from the Piwi-mediated pathway for TE regulation [34].

The proposal that Tex19.1 functions in an independent TE regulatory pathway is further supported by the finding that in murine placenta, where there are alterations to expression levels of some TEs, *Tex19.1* is the only known methylation-sensitive genome defense gene that is highly expressed [32], suggesting it may independently serve to protect placental cells from elevated TE expression [35]. Female *Tex19.1* ^*-/-*^ mutant mice also exhibit impaired placental function [33, 35]. Unlike *Tex19.1* ^-/-^ male testis tissue, *Tex19.1* ^-/-^ placental tissue exhibits elevated *LINE* expression and also exhibits some differential expression of protein coding genes [35]. Collectively, these findings suggest that Tex19.1 controls transcription/transcript related mechanisms to protect the germline and placental genomes [29, 33–35].

The finding that human *TEX19* is a CT gene opens the question of whether *TEX19* expression is oncogenic or provides a functional advantage to cancer cells [26, 31]. Expression of germline genes has been linked to poor patient prognosis in cancers, such as lung cancer (for example, see [36]), so revealing functional roles of these genes, if any, is important to understand the mechanisms of cancer development/progression. In this study we identify a requirement for TEX19 in human cancer cells to drive proliferation that reveal it to be a potential cancer-specific drug target and prognostic indicator.

## Methods

### Cell culture and proliferation/self-renewal assays

Human cacner cell lines used in this study are provided in Additional file 1; Table S1. Cells were cultured in McCoy’s 5A medium (Thermo Fisher Scientific, Runcorn, UK) supplemented with 10% fetal bovine serum (FBS; Life Technologies) or in RPMI medium supplemented with 10% FBS and 2 mM sodium pyruvate (Thermo Fisher Scientific, Runcorn, UK); SW480 cells were cultured in Dulbecco’s modified Eagle’s Medium (Thermo Fisher Scientific, Runcorn, UK).. All cells were cultured at 37 °C in 5% CO_2_ in a humidified incubator. All cells were authenticated once every 12 months using LGC Standards Cell Line Authentication service (last report number: 710236782; Teddington, UK). Cells were regularly checked for mycoplasma using the LookOut Mycoplama Detection Kit (Sigma, Irvine, UK).

For leptomycin B (LMB) treatment cells were seeded into 40 mm tissue culture dishes and grown to the required density. Cells were then treated with 10 ng/ml LMB (L2913; Sigma, Irvine, UK) and incubated for a further 16 h.

Extreme limiting dilution analysis (ELDA) was performed as previously described [37, 38]. Briefly, sphere-derived cell were collected from 10 cm dishes and diluted into single cell suspension and plated at concentrations of 1000 to 1 cells per 100 μl SCM using repeats of defined experimental conditions in 96 well ultra-low attachment plates (Costar Corning; Sigma, Irvine, UK). Cells were incubated at 37 °C in a 5% CO_2_ atm for 10 days. Cells were supplemented with 50 μl of stem cell media (SCM) and transfection complexes re-applied after 4 and 8 days of incubation. At the end of 10 days the number of wells showing spheres with more than 20 cells were counted by light microscope. ELDA web tool (hrrp://bioinf.wehi.edu.au/software/elda) was used to determine frequencies of sphere forming cells.

Staining for senescence was carried out using β-galactosidase Staining Kit (Cell Signaling, Leiden, Holland) following the manufacturer’s instructions.

### siRNA transfection

siRNAs used in this study are TEX19 siRNA A (5′-AGGATTCACCATAGTCTCTTA-3′), TEX19 siRNA B (5′-TTCAACATGGAGATCAGCTAA-3′) and a negative control (Qiagen, Manchester, UK, Allstars Negative Control siRNA). Transfection was carried out with HiPerFect (Qiagen, Manchester, UK) following the manufacturer’s instructions. Briefly, 150 ng of siRNA was mixed with 6 μl of HiPerFect and 100 μl of cell specific medium. This mix was incubated at room temperature for 15 min to permit transfection complexes to form and was then added in a dropwise fashion to approximately 1.5 × 10^5^ cells. The number of siRNA treatments per cell culture was dependent upon the specific experiment and siRNA was added to cells at least once every 24 h for proliferation assays over extended periods. Depletion was verified by RT-qPCR and/or western blotting.

### Whole cell extraction, fractionation and western blotting

Whole-cell lysates were prepared using M-PER lysis buffer (Thermo Fisher Scientific, Runcorn, UK #78503), Halt Protease Inhibitor Cocktail (Thermo Fisher Scientific, Runcorn, UK) and Halt Phosphatase Inhibitor Cocktail (Thermo Fisher Scientific, Runcorn, UK). Approximately 30 μg of protein extract were used for western blotting (WB). Samples were mixed with 2X Laemmli Buffer (1:1) (Sigma, Irvine, UK; S3401) and boiled at 100 °C for 5 min prior to electrophoresis. Precision Plus Protein Dual Color Standards (BioRad, Watford, UK) was used as a protein ladder. NuPAGE Novex 4–12% Bis-Tris gels (Thermo Fisher Scientific, Runcorn, UK) were used and electrophoresis was carried out in NUPAGE MOPS SDS buffer (Thermo Fisher Scientific, Runcorn, UK) for 90 min at 120 v. Fast Western Blot Kit, ECL substrate (Thermo Fisher Scientific, Runcorn, UK) was used according to manufacturer’s instructions to detect the primary antibodies. Membranes were probed with primary antibodies in 10% dry milk/PBS/0.5% Tween 20. Incubation with secondary antibodies was performed at room temperature for 1 h, followed by a 10 min wash in milk solution and 3 additional 10 min washes in PBS/0.5% Tween 20 at room temperature. Antibody detection was performed using Pierce ECL Plus Western Blotting Substrate (Thermo Fisher Scientific, Runcorn, UK).

Subcellular fractionation was carried out as follows. Following harvesting cells were resuspended in hypotonic buffer [50 mM Tris-HCl (pH 7.4), 0.1 M sucrose, 1 mM AEBSF] and lysis buffer C (1% Triton, 10 mM MgCl_2_, 1 mM AEBSF) at 1:1 ratio. Following incubation on ice for 30 min tubes were spun at 6000 g for 2 min. Supernatant contained cytoplasmic proteins and the pellet was resuspended in lysis buffer N [50 mM Tris-HCl (pH 7.4), 100 mM potassium acetate, 1 mM AEBSF] to extract nuclear protein.

The following antibodies were used in this study: anti-TEX19 (R & D Systems, AF6319), 1:200 dilution for WB; anti-Lamin B1 (Abcam, AB16048), 1:1000 dilution for WB; anti-tubulin (Sigma, T6074), 1:8000 dilution for WB; anti-cleaved caspase-3 (Cell Signaling, Leiden, Holland; 9664), 1:1000 dilution for WB; cell cycle cocktail (anti-pCDK2, anti-Actin, anti-pH3) (Abcam, Cambridge, UK; AB136810), 1:250 dilution for WB; anti-rabbit secondary antibody (Cell Signaling, Leiden, Holland; 7074), 1:3000 dilution for WB; anti-mouse secondary antibody (Cell Signaling, Leiden, Holland; 7076).

For the chromatin association assay (Ch) protein lysates were prepared consecutively with increased concentrations of NaCl. Protein extracts were subjected to western blotting as described using anti-a-tubulin and anti-histone H3 antibodies in addition to anti-TEX19 antibodies. 10 ng/ml KaryoMAX colcemid (Gibco, Runcorn, UK; 15212-012) was added to the growth medium to synchronize cells in metaphase prior to chromatin extraction.

### Reverse transcription quantitative polymerase chain reaction (RT-qPCR)

Total RNA was extracted from appropriate cell cultures using the RNeasy Plus Mini Kit (Qiagen, Manchester, UK) following the manufacturer’s instructions. First-strand cDNA synthesis was carried out using SuperScript III First-Strand Synthesis System (Thermo Fisher Scientific, Runcorn, UK) following the manufacturer’s instructions.

qPCR reactions were carried out using GoTaq qPCR Master Mix (Promega, Southampton, UK) in a CFX96 Real-Time PCR Detection System C100 thermal cycler (BioRad, Watford, UK). All RT-qPCR primers were obtained from Qiagen (Manchester, UK). The exceptions were the HERV primers and primers for *PIWIL3* and *PIWIL4. PIWIL3* primers were PIWIL3F (5′-TGGCATTGCATTAAGTAAGGG-3′) and PIWIL3R (5′-TTTGAAAAACGCAAACATCG-3′). *PIWIL4* primers were PIWIL4F (5′-CTGAAGGATACAGCGGGAAA-3′) and PIWIL4R (5′-AAAGATGCACTCAGCAAGGAC-3′). HERV primers are listed in Additional file 2; Table S2; other RT-qPCR primers are shown in Additional file 3; Table S3. Reactions were carried out in triplicate with PCR primers at a final concentration of 0.2 μM in a final volume of 25 μl. All PCR primers are available upon request. BioRad CFX Manager 2.0 software was used to determine primer efficiency/specificity, threshold cycle values (Ct values) and expression values using default parameters. Results were normalized using two or three reference genes and fold-change values were calculated based on the ΔΔCT method.

### RNA sequencing and data analysis

Total RNA was extracted using RNeasy Plus mini kit (Qiagen, Manchester, UK) according to the manufacturer’s protocol. RNA quality was checked on an Agilent Bioanalyzer RNA 6000 nano chip and was assessed to be of high quality (RIN > 9.8). Indexed sequencing libraries were then prepared using the Illumina TruSeq v2 protocol. Briefly, polyA-tailed RNA enriched on oligo-dT beads before fragmentation and random priming. Reverse transcription was carried out with second strand synthesis and the resultant double-stranded cDNA was end repaired, A-tailed and Illumina TruSeq adapters were ligated. Correctly ligated fragments were enriched by performing 12 cycles of PCR with primers complementary to the Illumina adapters. The final libraries were checked and quantified on the Agilent Bioanalyzer DNA 1000 chip and the Life Technologies Qubit High Sensitivity DNA assay system before being pooled to an equimolar concentration of approximately 10 nM. qPCR was performed on a 10^5^ dilution of the multiplex pool (Kapa Biosystems Library Quantification Kit; Sigma, Irvine, UK) before 12 pM of multiplex library was sequenced on one lane of an Illumina HiSeq (TruSeq v3 chemistry) generating 190 million reads passing filter. Reads were demultiplexed and fastq files generated using Illumina CASAVA v1.8.2 software.

Fastq data underwent guided alignment to the human genome (NCBI Build 37.2) using Tophat v2.0.6 [39] with default parameters. Read duplicates were removed using Picard (http://broadinstitute.github.io/picard) and counts per gene generated using HTSeq [40]. Differential expression at both the gene and exon level was carried out in R (https://www.r-project.org/) using the ‘DESeq’ and DEXSeq’ R packages [41]. The resulting *P* values were adjusted for multiple testing with Benjamini and Hochberg’s [42] method to control the false discovery rate. Genes with a *P* value <0.05 and a log_2_-fold change > 1.0 have been selected as significant. Pathway and gene ontology (GO) analysis was carried out in R v3.2.3 (https://www.r-project.org/) using the ‘GOstats’ R package [43].

### Fluorescence activated cell sorting cell cycle analysis

Following trypsinization cells were fixed in 70% ethanol at 4 °C overnight. Fixed cells were treated with 0.5 mg/ml RNase A (Sigma, Irvine, UK) and stained with propidium iodide (500 nM, Sigma, Irvine, UK). Stained cells were analyzed using a Partec CyFlow Cube 8 and cell cycle analysis was carried out using FCS Express 4 software.

### Immunohistochemistry (IHC) and human tissue

Human tissue was obtained from patients following the guidelines of the North Wales Research Ethics Committee – West. All tissues were fixed in formalin, embedded in paraffin and prepared as 4 mm slices. Tumour/normal tissue arrays were obtained from the Cooperative Human Tissue Network (University of Virginia, USA). Staining was automated using the Ventana Benchmark XT instrument. Chromogenic reactions were carried out using 3,3′-diaminobenzidine and slides were counter stained with haematoxylin. The rabbit polyclonal anti-TEX19 antibody (Abcam, Cambridge, UK; 185507) was used for TEX19 staining. Secondary antibody only staining was used as a control. Antigen retrieval consisted of 4 min wash with protease I. All slides were scanned using and Axio Scanner.Z1 Scanner (Zeiss Cambridge, UK).

### Tissue/cell immunofluorescence imaging

For staining of cultured cells 10^5^ cells were seeded on a cover slip in a 24-well plate with appropriate medium and grown to the required density. Cells were fixed in 4% paraformaldehyde in PBS for 10 min at room temperature and then washed three times with PBS at room temperature. Cells were incubated for 1 h in 5% FBA/0.3% Triton in PBS at room temperature. Cells were incubated with primary antibodies in 1% BSA/0.3% Triton in PBS at appropriate concentrations overnight at 4 °C. Following three 5 min washes in PBS appropriate concentrations of the required secondary antibody were incubated with the cells in the same buffer for 1 h at room temperature in the dark. Following final washing cover slips were mounted on slides with Vectashiled Hard Set Antifade Mounting Medium (Vector Laboratories, Peterborough, UK) and counter stained with DAPI (Sigma) as required. Images were acquired using a Zeiss LSM 710 confocal microscope and analyzed using ZEN software (Zeiss, Cambridge, UK).

For tissue staining 4 μM paraffin embedded section were deparaffinised and re-hydrated as follows: three times through xylene, two times through 100% ethanol, two times through 70% ethanol, two times through sterile distilled H_2_O. Antigen retrieval was performed by heating samples in boiling 10 nM sodium citrate buffer (pH 6.0) for 10 min and cooling slides to room temperature. Slides were washed in sterile distilled water and stained as for the cultured cells (see above).

Antibodies used for staining were as follows, anti-TEX19 (Abcam, Cambridge, UK; AB185507), dilution of 1:50; anti-MAGE-A1 (LSBio, Nottingham, UK; LS-C87868), dilution of 1:20; anti-vimentin (LSBio, Nottingham, UK; LS-B7191), dilution of 1:100; secondary goat anti-rabbit (Alexa Fluor 488; Thermo Fisher Scientific, Runcorn, UK; A11034), dilution of 1:1,1000; secondary goat anti-mouse (Alexa Fluor 568; Thermo Fisher Scientific, Runcorn, UK; A11031), dilution of 1:1000.

### Mouse tumourgenicity assay

In vivo tumour growth capability of Tex19shRNA-SW480-c3 cells was assessed by sub-cutaneous xenograft into immune deficient NSG mice (Envigo, Derby, UK). Cells were harvested using 1 mM EDTA and re-suspended at a density of 5 × 10^7^ cells/ml in serum free DMEM medium. A total of 5 × 10^6^ cells were injected sub-cutaneously into the flank of each mouse. Cells were allowed to establish for 6 days prior to induction of shRNA expression with doxycycline. To induce shRNA expression, mice were injected with 10 mg/kg intra-peritoneally every 2 days. Mice were then monitored, and when palpable, tumour volume was measured twice weekly with a digital caliper. Relative tumour volume (RTV) was calculated using xy^2^/2; where x is the longest axis of the tumour and y is the shortest axis of the tumour.

### Survival analysis

Cancer data sets available from The Cancer Genome Atlas (TCGA; http://cancergenome.nih.gov) were analyzed using R v3.2.3 (https://www.r-project.org/) to assess the association of *TEX19* expression and clinical data. Normalized gene RSEM values for all TCGA RNA-seq data sets as well as corresponding clinical data were downloaded from http://firebrowse.org. The survival analysis was carried out on primary tumour samples apart from leukaemia, where the primary blood derived cancer samples from peripheral blood were used. Normalized RSEM values were transformed to log_2_ counts per million prior to survival analysis using voom [44] in the ‘limma’ R package [45]. The patients were categorized in to two groups, low and high *TEX19* expression in cancer, split by the median value. Overall survival (in years) related to *TEX19* expression was computed with the Kaplan-Meier method and compared by the log rank test using the ‘survival’ R package [46]. *P* values of <0.05 were considered statistically significant.

## Results

### TEX19 is required for the proliferation and self-renewal of human cancer cells

**Fig. 1:**
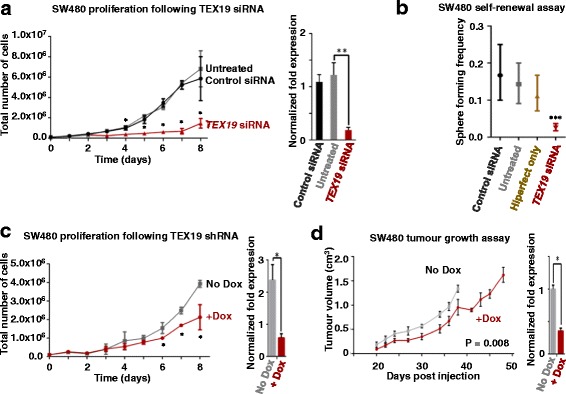
TEX19 is required for cancer cell proliferation and self-renewal: **a** Left hand line plot: siRNA-mediated (siRNA A) *TEX19* mRNA depletion results in inhibition of SW480 cancer cell proliferation (* ≤ 0.05; unpaired *t*-test); right hand bar chart: RT-qPCR analysis of *TEX19* mRNA levels for the cell cultures shown in the adjacent cell count plot (** ≤ 0.01; unpaired *t*-test; siRNA treatment occurred every 24 h and RNA extraction for RT-qPCR was taken 24 h following final siRNA treatment). **b** Extreme limiting dilution assay shows that *TEX19* siRNA treatment (siRNA A) results in loss of self-renewal capacity in SW480 cancer cells (*** ≤ 0.001; chi square test). Sphere formation was monitored following 10 days incubation. **c** Left hand line plot: shRNA-mediated (+dox) *TEX19* mRNA depletion results in reduction in proliferative potential of SW480 cancer cells (* ≤ 0.05; unpaired *t*-test; RNA extraction for RT-qPCR was taken 24 h following final Dox treatment); right hand bar chart: RT-qPCR analysis of *TEX19* mRNA levels for cell cultures shown in the adjacent plot (* ≤ 0.05; unpaired *t*-test). **d** Left hand line plot: SW480 cell carrying a dox-inducible shRNA cassette generate tumours with reduced volume when the mice are fed with dox (red line) compared with no dox (grey line). *P* value is derived from a Wilcoxon matched pairs test (2-tailed); right hand bar chart: RT-qPCR analysis of *TEX19* mRNA levels within final tumours extracted from mice used in the experimental set shown in the adjacent line plot (* ≤ 0.05; unpaired *t*-test: RNA was extracted for RT-qPCR analysis immediately following termination and tumour extraction for both +/- Dox)

Given the finding that *TEX19* is expressed in a range of cancers (http://cancergenome.nih.gov/) [26] and the finding that other CT genes play a role in oncogenesis (for example, see [13]), we set out to determine whether TEX19 contributes to proliferative potential of human cancer cells. We chose colon cancer cell line SW480 as our primary model system as these cells are enriched for cancer-stem-like cells. Other cells lines previously shown to express *TEX19* [26], including an additional colon line (HCT116) as well as a lung cancer line (H460) and an embryonal cancer line (NTERA2), were also employed to assess potential universality of proliferative and/or functional role(s) for TEX19. In these cell lines siRNA-depletion of TEX19 (using independent siRNAs) results in an inhibition of proliferation (Fig. 1a; Additional file 4: Figure S1a-d; siRNA-mediated TEX19 depletion was confirmed by RT-qPCR and western blotting, examples given in Fig. 1a; Additional file 4: Figure S1; western blots also validate the anti-TEX19 antibody).

*TEX19* expression is found in germ cells/ESCs, and murine Tex19.1 has been linked to ESC self-renewal [33]. To determine whether human TEX19 functions in self-renewal in cancer stem/progenitor cells, we carried out extreme limiting dilution assays (ELDA) to determine self-renewal ability in sphere-derived SW480 cells and NTERA2 cells [37]. Cultures treated with independent siRNAs had reduced sphere formation from single cells (Fig. 1b; Additional file 5: Figure S2). This demonstrates that TEX19 is required for proliferation/self-renewal of cancer cells, which could infer such a role in germ and stem cells. Over expression of *TEX19* in SW480 cells using a doxycycline inducible *TEX19* did not further accelerate proliferation (Additional file 6: Figure S3), suggesting the requirement for TEX19 may have an upper threshold.

To determine whether TEX19 plays a role in self-renewal/proliferation in tumours in vivo we developed SW480 and HCT116 cell lines carrying an inducible shRNA cassette. A clone (*TEX19*shRNA-SW480-c3) was selected and proliferation analysis with and without doxycycline (dox) was carried out (expressing and not expressing *TEX19*shRNA). Induction of the shRNA resulted in a moderate, but statistically significant reduction in proliferation (Fig. 1c). A similar proliferative inhibition was observed with shRNA induction in HCT116 (Additional file 7: Figure S4). The inducible-shRNA SW480 cells were injected subcutaneously into NSG mice. One cohort was injected with dox, whereas a control population was not. Despite the relatively mild proliferative reduction observed in vitro, TEX19-shRNA induction in mice resulted in significant reduction of tumour volume, indicating that *TEX19* expression is required for tumour development (Fig. 1d).

**Fig. 2:**
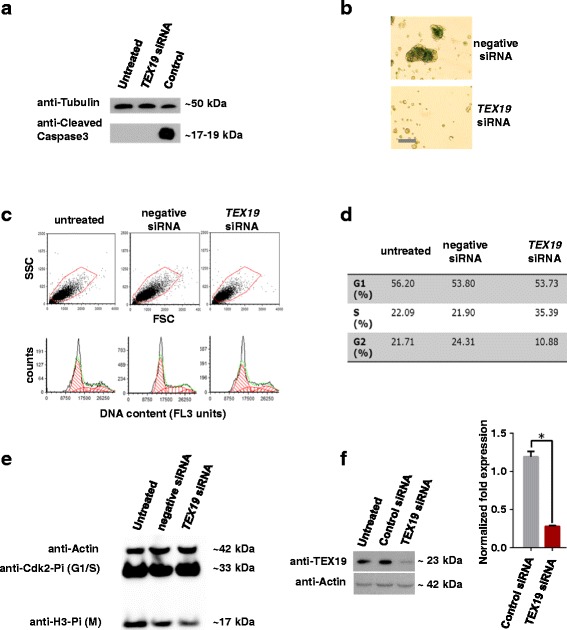
Analysis of TEX19 depleted SW480 cells: **a** TEX19 depleted SW480 cells do not have cleaved caspase-3 indicting there is no measurable apoptosis. **b** TEX19 depleted SW480 cells (lower image) do not form full spheres and exhibit no staining with β-galactosidase indicating that they are not undergoing lysosome-dependent senescence (upper image shows SW480 spheres in the absence of TEX19 siRNA staining positive for β-galactosidase due to natural senescence of internal cells; bar = 250 μm). **c** FACS of TEX19 siRNA treated SW480 cell populations (a minimum of 10,000 cells were analyzed in each case). **d** TEX19 siRNA treated cells have a lower proportion of the population in G2 and a higher proportion in S phase. **e** Western blot of whole cell extracts of SW480 cells depleted for TEX19 have lower levels of phosphor-histone H3 indicating fewer cells in mitosis. **f** Western blot and RT-qPCR analysis demonstrating TEX19 depletion (for data shown in **a** and **c**-**e**)

To gain insight into the role that TEX19 plays, we assessed various parameters of cells depleted for TEX19. Whilst proliferation of SW480 cells is inhibited following TEX19-depletion, the cells do not stain with trypan blue, suggesting that they retain viability, at least in the short-medium term. We next tested whether these cells become apoptotic. This was assessed by western blot analysis of cleaved caspase-3, a marker of the caspase-dependent apoptotic programme. No measurable level of cleaved caspase-3 was detected (Fig. 2a), consistent with murine *Tex19.1* ^-/-^ ESCs [33]. Taken together these two observations indicate that TEX19-depleted cells remain viable and, unlike murine *Tex19.1* ^-/-^ spermatocytes, are not undergoing measurable programmed cell death [33, 34]. Next we tested siRNA treated cells from the ELDA with β-galactosidase, a measure of lysosome-dependent senescence. No measurable staining was observed, indicating these cells do not appear to be senescent (Fig. 2b). However, when TEX19-siRNA treated cells that failed to proliferate were washed and placed in siRNA-free fresh media, they did not re-initiate proliferation, suggesting that they have entered a non-senescent, non-apoptotic state from which they cannot readily recover, possibly a quiescent-like state.

To assess the cell cycle status of TEX19-depleted cells we analysed DNA content using FACS (Fig. 2c, d). The percentage of cells with a 2C compliment (G1) appears to remain unaltered. However, more cells accrue in S-phase and there are fewer cells with a full 4C compliment (G2) indicating that TEX19-depleted cells appear to have a delay in S-phase progression, albeit limited. We employed western blots on whole cell extracts of TEX19-depleted cells using antibodies to assess cell cycle progression [anti-phopho CDK2 Tyr15 (G1-S transition), anti-phopho histone H3 Ser10 (mitosis), anti-actin (loading control)] (Fig. 2e). There was no notable change in phospho-CDK2, supporting the FACS data, which indicate these cells progress normally into S-phase. There is an apparent reduction in the levels of phopho-histone H3 indicating there are fewer cells entering mitosis, again, consistent with the FACS profile which shows a limited delay in S-phase progression/completion. Collectively, these data indicate that whilst TEX19-depleted cells are viable (in the short-term, at least), the inhibition of proliferation might be linked to a delayed progression through S-phase which does not trigger apoptosis or senescence.

### TEX19 is present is both the nucleus and the cytoplasm of human cancer cells

**Fig. 3:**
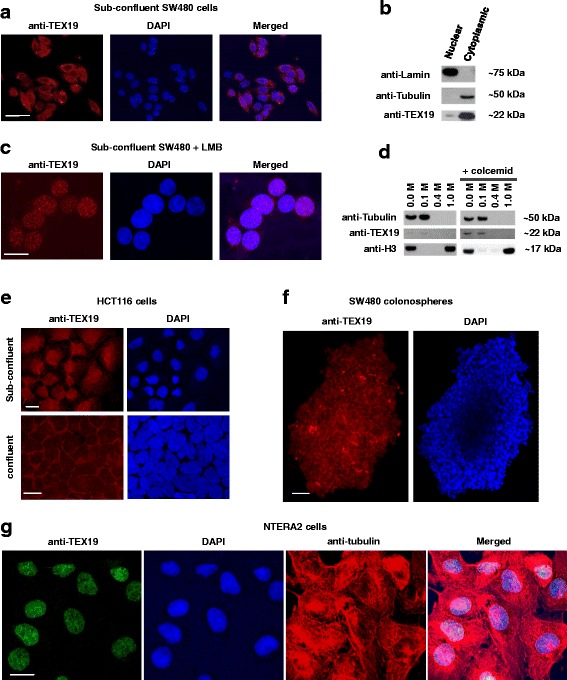
TEX19 has nuclear and cytoplasmic localization in cancer cells: **a** Sub-confluent SW480 cells show TEX19 (red) localized to both nucleus and cytoplasm. Distinct cells show distinct proportions of cellular TEX19 within the nucleus. DNA is stained with DAPI (blue); bar = 40 μm. **b** Western blot analysis of nuclear and cytoplasmic extracts of sub-confluent SW480 cells indicates the majority of TEX19 is cytoplasmic, but a clear nuclear fraction is detected. Lamin (nucleus) and tubulin (cytoplasm) are used as controls (controls show clean faction signals, indicating no inter-fraction contamination). **c** Sub-confluent SW480 cells treated with the nuclear export blocking agent LMB demonstrates that TEX19 (red) can accumulate in the nucleus. DNA is stained by DAPI (blue); bar = 20 μm. **d** Western blot analysis of chromatin associated TEX19 demonstrates that TEX19 does not have a tight association with chromatin, even when cells are blocked in M phase with colcemid. Histone H3 is a control for tight chromatin association (only dissociated with 1.0 M NaCl); tubulin is mostly cytoplasmic (see b) and does not associate with chromatin during the chromatin preparation. **e** TEX19 is predominantly cytoplasmic at higher cell densities. Staining of sub-confluent HCT116 cells (top panels) indicates that TEX19 (red; blue = DAPI) is nuclear and cytoplasmic at this cell density, as for SW480 cells. When cells reach confluence (lower panels) the majority of TEX19 is cytoplasmic. Bar = 20 μm. **f** When SW480 cells are densely associated in spheres TEX19 (red; DAPI = blue) is mostly cytoplasmic. Bar = 100 μm. NTERA2 cells show strong nuclear staining with anti-TEX19 antibodies (green) irrespective of level of confluence. Red = anti-tubulin; Blue = DAPI; bar = 20 μm. **g** NTERA2 cells exhibit predominantly nuclear TEX19. Green = anti-TEX19; Blue = DAPI; Red = anti-tubulin; bar = 20 μm

To gain insight into the possible function(s) of TEX19 in cancer cells, we assessed cellular localization. Previous studies on murine cells have reported a predominantly cytoplasmic localization for Tex19.1 [33, 34], but with some nuclear Tex19.1 in placental cells [35]. Immunostaining of TEX19 in sub-confluent SW480 cells showed TEX19 located in both the cytoplasm and the nucleus, with some cells having a stronger nuclear staining than others (Fig. 3a). To further verify that there was a nuclear fraction of TEX19, we carried out western blots on nuclear and cytoplasmic extracts, which demonstrated that the majority of TEX19 is cytoplasmic in sub-confluent SW480 cells, but there is a measurable amount of TEX19 in the nucleus (Fig. 3b). To further verify the finding that some TEX19 is nuclear, we treated SW480 cells with leptomycin B (LMB), an inhibitor of the CRM1-dependent nuclear export pathway. LMB treated cells have a clear accumulation of TEX19 in the nucleus (Fig. 3c). Interestingly, the nuclear TEX19 in LMB treated cells forms clear foci, potentially indicating that TEX19 is forming region-specific complexes within the nucleus. These foci are consistent with chromatin association, so we isolated chromatin from sub-confluent SW480 cells and treated it with increasing concentrations of salt. TEX19 dissociated from the chromatin fraction at low salt concentrations indicating it does not have a strong association with chromatin (under these conditions; Fig. 3d). To determine whether nuclear localization of TEX19 is a common feature of cancer cells, we stained a second cell line, H460 (lung carcinoma). As for SW480 cells, sub-confluent H460 cells have nuclear and cytoplasmic TEX19 (Fig. 3e). We noticed, however, when cells were grown to over-confluence that TEX19 becomes excluded from the nucleus and appears to be solely cytoplasmic (Fig. 3e). To expand this observation, we grew colonospheres from SW480 cells, which are enriched for cancer stem-like cells [38], and assessed TEX19 localization. In these spheres, where cells are in close contact, TEX19 is predominantly cytoplasmic (Fig. 3f), consistent with the majority of Tex19.1 in murine tissue [33, 34]. Remarkably, in the germline tumour cell line, NTERA2, anti-TEX19 staining is predominantly nuclear (Fig. 3g), which could infer a link to germline/stem capabilities for TEX19.

### TEX19 localization in human testis and cancer tissues

**Fig. 4:**
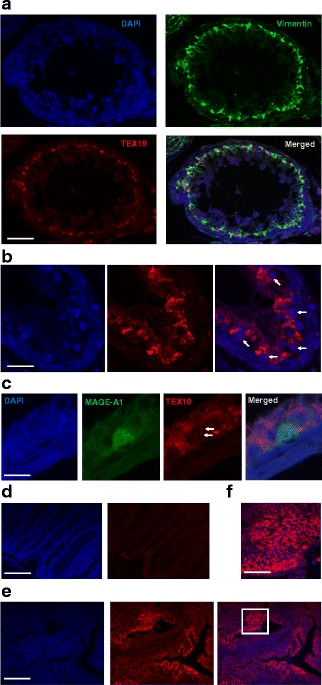
TEX19 is predominantly localized to the cytoplasm in normal testis and cancer tissues: **a** Staining of a human testicular seminiferous tubule indicates that TEX19 (red – bottom left panel) is predominantly located in the basal layer of cells and is mostly cytoplasmic. Co-staining with an anti-vimentin antibody (green – top right), which marks Sertoli cells, reveals that TEX19 staining is adjacent to, but not overlapping with vimentin. Bar = 50 μm. **b** Nuclear foci are apparent when testis are stained with anti-TEX19 (red; blue = DAPI) antibody. White arrows in the merged image show the localization of the nuclear staining with anti-TEX19 antibodies (red; DAPI = blue). **c** Co-staining with anti-MAGE-A1 antibodies (spermatogonial cells; green) and anti-TEX19 antibodies (red; blue = DAPI) indicate that TEX19 nuclear foci are associated with spermatagonia. White arrows indicate anti-TEX19 stained nuclear foci. Bar = 10 μm. **d** Staining of clear margin morphologically normal colon tissue taken from a cancer patient indicates that there is no staining with anti-TEX19 antibody (red; right hand panel; blue = DAPI, left hand panel). Bar = 100 μm. **e** Staining of matched (to tissue shown in D) colon tumour material shows regions of intense staining with anti-TEX19 antibodies (red; blue = DAPI). Bar = 100 μm. **f** Enlargement of the colon cancer region of intense anti-TEX19 signal (white box; scale bar is 30 μm) shows that anti-TEX19 staining (red) is mostly cytoplasmic

Our finding that human cancer cells have both nuclear/cytoplasmic TEX19, led us to investigate the localization in human cancerous tissues. Previous analysis of human TEX19 by immunohistochemistry (IHC) demonstrated that it is not present in normal healthy non-testis tissues, however, analysis of human testes lead to the proposal that TEX19 was located in seminiferous tubule Sertoli cells [31]. This, seems to be in conflict with a direct role for TEX19 in germ cells / stem cells. To explore this further we used immunofluorescence to co-stain human testes with anti-TEX19 and anti-vimentin antibodies; (vimentin is a Sertoli cell marker). TEX19 staining is located to the basal layer, which mostly consists of spermatagonial cells and basal sections of Sertoli cells (Fig. 4a) [47], indicating that TEX19 staining is in specific cells or specific sub-cellular regions. Co-staining with an anti-vimentin antibody reveals that the high intensity TEX19 staining, whist in close proximity to the vimentin stain, does not directly co-localize (Fig. 4a), possibly indicating that TEX19 is present in a sub-compartment of Sertoli cells or in cells closely associated with Sertoli cells. There does, however, appear to be a low intensity TEX19 staining that that is quite extensive throughout the basal regions, including Sertoli cells, suggesting distinct TEX19 positive regions within the basal areas of seminiferous tubules. Whilst localization was mostly cytoplasmic, we observed foci within large nuclei of cells located within the basal layer of the tubules, possibly spermatagonia (Fig. 4b). Spermatagonia express *MAGE* genes (known CT genes), so we co-stained tubules with anti-MAGE-A1 antibodies. This appears to show TEX19 foci only within the nuclei of MAGE-A1 positive cells (Fig. 4c). This indicates that there are distinct cellular and sub-cellular TEX19 fractions within the testis, consistent with cancer cells.

We next determined the subcellular localization of TEX19 in cancerous tissue. We used colon cancer tissue and matched histologically non-cancerous adjacent tissue [48]. Immunofluorescence revealed that adjacent, clear margin tissue with a morphologically normal appearance did not stain positive for TEX19 (Fig. 4d). The cancerous tissue, however, had regions of strong TEX19 staining (Fig. 4f) demonstrating that *TEX19* encodes an antigen within colorectal cancer tissue. Cancerous tissue has heterogeneous regions that have high levels of TEX19 or none/low levels of TEX19, which fits with emerging models of tumour heterogeneity [49, 50]. The regions of intense TEX19 staining reveal that staining appears to be mostly cytoplasmic (Fig. 4f), consistent with highly confluent cancer cells and the majority of closely associated cells within colonospheres (Fig. 3). We cannot, however, exclude the possibility of low levels of nuclear TEX19 in these cells and we did observe occasional TEX19 foci associated with DAPI stained nuclear material in the tumour tissue (Fig. 4f).

### TEX19 is associated with early adenoma stages of colorectal cancer development

**Fig. 5:**
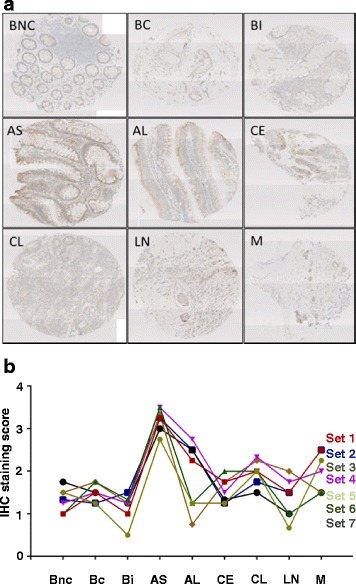
TEX19 is associated with early adenoma stage of colorectal cancer: **a** IHC showing an example of a colorectal cancer tissue progression array stained with anti-TEX19 antibody. BNC = non-neoplastic colonic mucosa from pre-cancer case; BC = non-neoplastic colonic mucosa from cancer case; BI = inflamed non-neoplastic mucosa; AS = colonic adenoma, < 2 cm in diameter; AL = colonic adenoma, > 2 cm in diameter; CE = invasive adenocarcinoma, stage T1/T2; CL = invasive adenocarcinoma, stage T3/T4; LN = colorectal adenocarcinoma metastatic to lymph node; M = colorectal adenocarcinoma metastatic to distant sites. 10 X magnification used for all images shown. **b** Analysis of seven independent colorectal cancer tissue progression arrays stained with anti-TEX19 antibody. 0 = no staining; 1 = very weak/background staining; 2 = weak staining; 3 = strong staining; 4 = very strong staining

To determine the stage of tumour progression at which TEX19 becomes apparent, we used IHC to stain colorectal cancer progression arrays (Fig. 5a). We analysed seven independent colorectal tumour progression arrays. TEX19 was present in all arrays with highest staining observed in the early adenomas (<2 cm), with different levels present in later stage disease tissues (Fig. 5b). This observation indicates that in colorectal cancer development TEX19 is present at an early stage in all array series tested, potentially suggesting a requirement for TEX19 at an early phase.

### TEX19 regulates distinct transposable element transcripts in a cell-specific fashion

Previously, it has been demonstrated that murine Tex19.1 represses transcript levels from some endogenous retroviruses (e.g., *MMERVK10C*) during spermatogenesis, but not other TE transcripts (including *LINE*s, *SINE*s and other endogenous retro elements), suggesting that it can differentially regulate TE transcript levels. In contrast, in *Tex19.1* ^-/-^ murine placental tissue, where Tex19.1 regulates intra-uterine growth, distinct TEs, such as *LINE*s are up-regulated, whereas expression of the endogenous retrovirus *MMERVK10C*, which is repressed by Tex19.1 during spermatogenesis, remains unaltered [34, 35]. Murine *Tex19.1* ^-/-^ ESCs exhibit yet another pattern of TE expression, with up-regulation of *MMERVK10C* and other TEs, such as *LINE-1* [33]. These findings indicate that whilst Tex19.1 regulates TE transcript levels in the mouse, there is heterogeneity that might reflect a tissue or cell type distinction in Tex19.1 function.

**Fig. 6:**
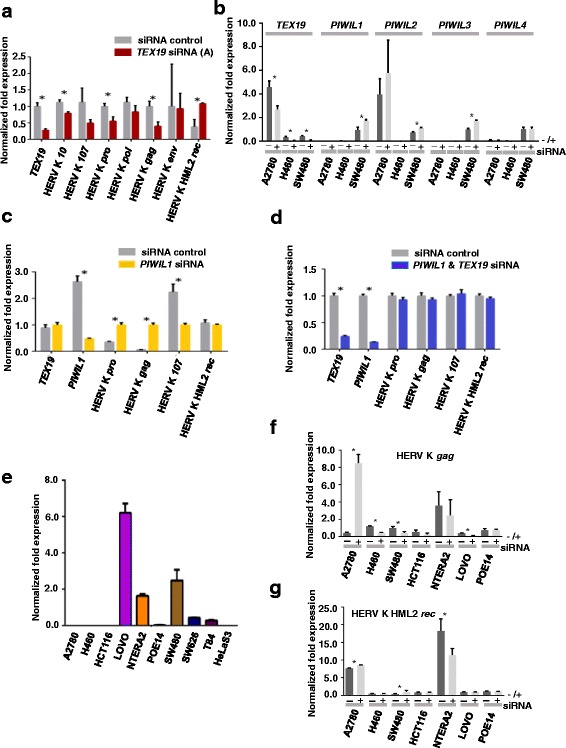
Differential TEX19-assocaited control of TE and piRNA regulatory genes in distinct human cancer cell lines: **a** RT-qPCR analysis of HERV genes in SW480 cells depleted for *TEX19* mRNA indicates that TEX19 regulates HERV gene expression. **b** RT-qPCR analysis of expression of piRNA regulator genes (*PIWIL1-4*) in three cancer cell lines (A2780, H460, SW480) depleted for TEX19 mRNA indicates that whilst TEX19 controls some, but not all piRNA genes (*PIWIL1-3*) in SW480, this is not a universal feature of TEX19 in all cancer cell lines. **c** RT-qPCR analysis of HERV genes in SW480 cancer cells depleted for *PIWIL1* mRNA indicates that PIWIL1 regulates HERV gene expression. **d** RT-qPCR analysis of SW480 cancer cells depleted for both *TEX19* and *PIWIL1* mRNA demonstrates that TEX19 regulates HERV gene expression in SW480 cells via a PIWIL1-dependent pathway. **e** RT-qPCR analysis of mRNA extracts from a range of cancer lines indicates that unlike *TEX19*, expression of *PIWIL1* is not universal in these cancer cell lines indicating that a functional link between TEX19 and PIWIL1 is not universal. **f** RT-qPCR analysis of HERV *gag* gene in seven cancer cell lines depleted for *TEX19* mRNA (+/- siRNA = +/- *TEX19* siRNA). **g** RT-qPCR analysis of HERV *rec* gene in seven cancer cell lines depleted for *TEX19* mRNA (+/- siRNA = +/- *TEX19* siRNA)

TE activation occurs during oncogenesis [51, 52]. Given that human TEX19 might regulate progression through S-phase (see above), and the role for murine Tex19.1 in TE control, it is not unreasonable to postulate that elevated transposition or TE expression caused by depletion of TEX19 might retard S-phase. Given this, we used RT-qPCR to determine whether depletion of TEX19 resulted in alteration in TE RNA levels in SW480 cells. As for murine spermatogenesis, we found no change in *LINE* or *SINE* transcript levels. We did, however, find that some HERVK transcripts were altered; unexpectedly, some were down-regulated (e.g., HERVK *pro*), but HERVK *HML2 rec* was up-regulated (Fig. 6a). This demonstrates that human TEX19 differentially controls HERVK transcripts in SW480 cells. The reduction in some transcripts was a little unexpected as murine Tex19.1 appears to function in TE transcript suppression [34]. Given that we saw reductions in transcripts, and given the fact that murine Tex19.1 also controls expression of protein encoding genes in the placenta, we questioned whether reduction of human TEX19 might activate the human PIWI pathway, which may then reduce some HERVK transcripts. To assess this, we determined expression levels of human *PIWI* orthologues (*PIWIL1-4*). All four were expressed in SW480 cells and *PIWIL1-3* are up-regulated following TEX19 depletion (Fig. 6b; *PIWIL4* was not).

To test whether PIWI pathway activation could reduce levels of some HERVK transcripts, we carried out single and double gene siRNA depletion of TEX19 and PIWIL1 in SW480 cells. Depletion of PIWIL1 alone results in both an elevation and a reduction of HERVK transcripts (Fig. 6c), indicating a differential suppression and activation role for PIWIL1. Remarkably, co-depletion of PIWIL1 and TEX19 results in no measurable change to HERVK transcript levels, suggesting that the HERVK transcript level changes following depletion of TEX19 in SW480 cells could be PIWIL1-dependent (Fig. 6d). However, if this was simply due to PIWIL1 acting downstream of TEX19, co-depletion of TEX19 and PIWIL1 should result in a PIWIL1-difficient phenotype (e.g., elevation of HERVK *pro* transcripts), which is not the case. Alternatively, this points to a more complex interplay between TEX19 and PIWIL1 in SW480 cancer cells, possibly indicating that TEX19 and PIWIL1 have opposing and independent roles in regulating TE transcripts (for example, TEX19 positively regulates HERVK *pro*, whereas PIWIL1 negatively regulates HERVK *pro* transcripts). This model is supported by the observation that HERVK *gag* transcripts (for example) are reduced following TEX19 depletion in H460 cells, which do not exhibit measurable expression of *PIWIL1* (Fig. 6d). A model in which TEX19 functions independently of PIWI pathway proteins is consistent with observations in the mouse [34].

To assess the universality of this phenomenon, we explored *PIWIL1* expression in other cell lines (Fig. 6e). We could detect no expression in A2780, H460, HCT116 or HeLaS3 cells and virtually no expression in PEO14. In two of the negative lines, H460 and A2780, we analysed the expression of the other *PIWI* paralogues. We could detect no expression of paralogues in H460, with A2780 expressing *PIWIL2* and relatively low levels of *PIWIL4*; however, the levels of neither were significantly altered by TEX19 depletion, suggesting that TEX19 regulation of *PIWI* gene expression may not be universal in all cancer cell types (Fig. 6b).

We next addressed whether TEX19 influenced transcriptional activation of HERV genes in other cell lines, including H460 where no *PIWI* paralogues were expressed. These analyses indicate that there are some changes (for example, see Fig. 6f, g). For example, A2780 shows considerable activation of HERVK *gag* transcripts in response to a relatively modest depletion of TEX19 (Fig. 6f).

### TEX19 regulates protein coding gene transcript levels

To explore the possibility that TEX19 acts as a protein coding gene transcriptional regulator in cancer cells, we carried out RNA sequencing on total polyA-RNA extracted from untreated SW480 cells and cells treated with a *TEX19* siRNA. Transcript levels from 80 genes were found to be significantly (*P* < 0.05) altered (up-/down-regulated), including a reduction in *TEX19* mRNA, as expected, and an increase in *PIWIL1* mRNA, consistent with our RT-qPCR (Additional file 8; Figure S5). Of the 80 genes, some are known CT genes; for example, we previously reported *SEPT12* and *RAD21L1* as CT genes [26], so these genes require TEX19 to control their expression in SW480 cells. Given that we found that *PIWIL1* expression regulation is not uniform in all cell lines, we carried out RT-qPCR for 52 of these genes (those with an RNA-seq *P* value of <0.01; Additional file 3; Table S3) in additional cell lines (H460, A2780, PEO14, NTERA2, and an independent duplicate analysis of SW480). No genes exhibited consistent changes across all cells lines (Additional file 3; Table S3), and distinct analysis of SW480 gave some inconsistencies suggesting a variability due to TEX19 dose (independent siRNA depletion of TEX19 is unlikely to have been uniform in all experiments). However, many gene transcripts did exhibit changes (up or down) indicating that TEX19 can regulate transcript levels of protein coding genes, possibly in a TEX19 dose-dependent fashion. Some of the genes, for example, *TSN* and *MYB*, are potential regulators of proliferation [53–57] and so this could indicate that TEX19 is required by cancer cells to drive proliferation by maintaining sufficient levels of oncogenic transcripts.

### High TEX19 expression is a prognostic marker in a range of cancer types

**Fig. 7:**
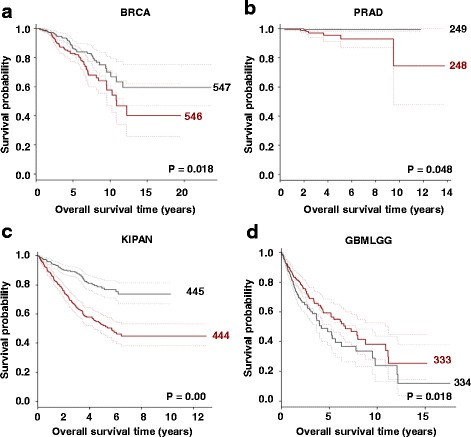
*TEX19* expression is linked to cancer prognosis: **a** Kaplan-Meier (KM) plot showing overall survival (OS) for breast cancer patient cohort (BRCA) indicates that higher *TEX19* expression is linked to a poor prognosis. Populations are divided by median *TEX19* expression (red = high; grey = low). Dashed lines are 95% confidence intervals. **b** KM plot visualizing OS for a pan-kidney (KIPAN) cancer patient cohort indicates that high *TEX19* expression is linked to a poor prognosis. Populations are divided by median *TEX19* expression (red = high; grey = low). Dashed lines are 95% confidence intervals. **c** KM plot showing OS for a prostate (PRAD) cancer patient cohort indicates that high *TEX19* expression is linked to a poor prognosis. Populations are divided by median *TEX19* expression (red = high; grey = low). Dashed lines are 95% confidence intervals. **d** KM plot visualizing OS for a glioma (GBMLGG) cancer patient cohort indicates that high *TEX19* expression is linked to a better prognosis. Populations are divided by median *TEX19* expression (red = high; grey = low). Dashed lines are 95% confidence intervals

Expression of germline genes has previously been reported to be linked to poor prognosis in cancers, for example, lung cancer (for example, see [36]). Given the finding that TEX19 controls cancer cell proliferation we wished to explore whether *TEX19* expression influences clinical progression. We used RNA-seq data from TCGA to determine whether high levels of *TEX19* expression were linked to poor prognosis. For each cohort of cancer patients we divided the population into high and low *TEX19* expression groups split by the median value and performed survival analysis. 37 data sets for distinct cancer groups were available, of which 27 held sufficient data for analysis (TCGA; Additional file 9; Table S4). High *TEX19* expression (as split by the median) is significantly linked to overall survival in seven data sets. For breast invasive carcinoma (BRCA; Fig. 7a), prostate adenocarcinoma (PRAD; Fig. 7b) and the pan-kidney cohort (KIPAN; Fig. 7c) high TEX19 expression is associated with poor prognosis; although the difference is marginal for PRAD. The KIPAN data set consists of data from three distinct renal cancer data sets (kidney chromophobe – KICH, kidney renal clear cell carcinoma – KIRC, kidney renal cell carcinoma – KIRP). Analysis of two independent kidney cancer data sets (KIRK and KIRP) reveals that both show significant reductions of overall survival for the higher *TEX19* expression cohort of patients (Additional file 10: Figure S6a/b); KICH is a rare renal cancer, only contributing to approximately 5% of all renal cancers [58], and it has a better prognosis than more common forms of renal cancer [59], so limited data excluded it from individual analysis.

Intriguingly, higher *TEX19* expression is linked to a better prognosis for gliomas (GBMLGG; Fig. 7d) and brain lower grade glioma (LGG; Additional file 10: Figure S6c). This finding was unexpected as previous studies have indicated germ line gene expression is linked to a poor prognosis.

The use of a single criteria for the cohort split point, such as splitting by the median, limits analyses of this nature. Use of other split points resulted in additional cancer types showing *TEX19* expression linked to prognosis; two examples are the data sets for lung adenocarcinoma (LUAD) and cervical squamous cell carcinoma/endocervical adenocarcinoma (CESC) when patients are split into the upper 25% of *TEX19* expression *vs.* the lower 75% expression for cancers where clinical data are available, where a significant (*P* = 0.039 and *P* = 0.038 respectively) link to poor prognosis is observed [which is not significant (*P* = 0.136 and *P* = 0.236 respectively) when the split is by the standard method of the median] (Additional file 11; Figure S7). Increased data volume and further sub-categorization of tumour types is likely to further resolve which cancer types can be stratified by *TEX19* expression profiling, but these initial findings clearly indicate that *TEX19* expression is linked to distinct clinical outcomes. In many of the cell line experiments we conducted we used colon cancer cells, yet interestingly, none of the splits we allocated to colorectal cancer data (263 patients; 124 expressing *TEX19* at the threshold set for positive expression) indicated a link to prognosis. There could be number of reasons for this, not least of which is the heterogeneous nature of TEX19 distribution to distinct tumour regions in the colon tumour samples we analysed (i.e. the RNA-seq data may have been obtained from tumour biopsy regions that may have had limited/no *TEX19* expression); given this, we cannot rule out a link between *TEX19* expression and cancer progression for colorectal cancers. The distinct link of high *TEX19* expression to positive and negative prognosis is discussed further below.

## Discussion

The idea that cancer cells achieve self-renewal potential by re-activating programmes that regulate the germ/stem like state, and undergo a soma-to-germline transition is gaining traction (for example, see [2, 3, 5]). Germline functions are known to contribute to distinct biological features of cancers [11–13], including invasiveness/metastasis (for example, [21–23, 25]) and maintenance of proliferative potential (for example, [14–19]). Some activities are linked to poor outcomes; for example, elevated expression of *SPANX-A/C/D* is linked to poor prognosis in breast cancers [25]. Furthermore, germline gene expression in lung cancer has been linked with aggressive, metastases prone disease and can be used for stratification of patients to identify cohorts who might benefit from targeted therapies [36]. Even meiotic chromosome regulators have been shown to contribute to oncogenesis by driving inter chromosomal associations required for oncogenic alternative lengthening of telomeres [60]. So, germline/stem cell factors can make distinct contributions to cancer progression, maintenance and evolution.

### TEX19 as an oncogenic driver

Despite the growing evidence for the soma-to-germline transition of tumours, the reported roles of specific germline genes in cancers remains limited. Here we find that *TEX19* expression is required to maintain the proliferative potential of a range of different cancer cells. Given that *TEX19* is expressed in many cancer types, this might infer that it is an oncogenic factor, indeed, all cell lines / tumour samples we analysed showed evidence of *TEX19* expression. This is not the case for all database studies [for example, the Human Protein Atlas (www.proteinatlas.org) reports only limited *TEX19* expression], but we have demonstrated that some areas of later stage tumours are more prone to *TEX19* expression, suggesting that expression can be regional within an advanced tumour. It is known that tumours can evolve and acquire a high degree of intra- and inter-tumour heterogeneity, and so database samples may also include information acquired from tumour regions that do not express *TEX19* [49, 50]. The heterogeneous distribution of TEX19 indicates two possibilities; firstly, *TEX19* may be ‘on’ during the early stages of tumourigenesis and becomes deactivated during the evolution of the tumour, or, alternatively, *TEX19* only becomes activated in specific regions as the tumour develops and grows. The finding that germline genes are actually required for the early oncogenic process in *D. melanogaster* l(3)mbt tumours might suggest the former is the case [7]. Our analysis of colorectal tumour progression profiles (Fig. 5) supports this view, as TEX19 was detected in all early adenomas, but was not detected in some samples of later stages of tumour progression which could point to an early ‘on’ later ‘off’ model for *TEX19*. This suggests that therapeutic targeting of TEX19 might not eliminate all tumour cells in later stage tumours. However, given the fact that TEX19 function is implicated in stemness [29], it might be the case that the TEX19 positive cells are those that retain stem-like features and thus therapeutic targeting of these cells remains important, as these cells might be driving the poor prognosis/therapeutic resistance [1].

### How does TEX19 modulate proliferation?

We have demonstrated that *TEX19* expression is required to maintain proliferation/self-renewal in a range of cancer cell types. Loss of murine *Tex19.1* can result in spermatogenic cells entering apoptosis, however, analysis of TEX19-depleted human cancer cells indicates that they are likely to be in a quiescent-like state, which appears to be linked to a failure to proceed through S-phase with normal kinetics. This observation is in contrast to murine *Tex19.1* ^-/-^ ESCs, which, whilst being defective in self-renewal, do not exhibit any overt S-phase defects [33]. Murine Tex19.1 has been shown to control levels of TE transcripts and, in the case of the female placenta, other protein coding transcripts [35]. We have demonstrated here that TEX19 in SW480 cells can influence TE transcript levels. In SW480 cells this appears to be counteracted by a PIWIL1-dependent mechanism. A model in which TEX19 and PIWIL1 serve in opposing and independent mechanisms for TE transcript control is consistent with murine Tex19.1 acting on TEs in a PIWI-independent fashion [34]. Indeed, in other cancer cells lines we tested PIWIL1 is not expressed and yet TE transcripts exhibit a measurable change upon TEX19 depletion, which supports a TEX19-dependent, PIWI-independent pathway.

The ovarian carcinoma cell line A2780 showed considerable activation of HERVK *gag* transcript levels upon a relatively moderate reduction of TEX19. A2780 cells do not express measurable levels of *PIWIL1* and the *PIWI* orthologues that are expressed are not altered upon TEX19 depletion. This indicates that in A2780 cells TEX19 appears to operate in a PIWI-independent mechanism for TE suppression, similar to that proposed for murine placental TE regulation [35]. These findings also demonstrated that TEX19 can act in a repressive and activating fashion for some TE transcripts in a cell- and/or dose-dependent fashion.

Given this, we explored protein coding gene changes with an aim of identifying changes common to all cells. This showed that no gene transcripts were consistently altered upon TEX19 depletion*.* However, coding gene transcripts were altered (up and down) indicating that TEX19 could regulate the transcripts/transcription of a cohort of oncogenic protein coding genes to foster a proliferative state, possibly in a dose-dependent fashion.

SSX2, another CTA, is a chromatin regulator [61] and it has been inferred that it may regulate cancer cell proliferation through transcriptional regulation [13]. Also, the germline-specific chromatin regulator ATAD2 drives various cancer progression phenotypes via transcriptional regulation and is linked to poor prognoses in various cancers and offers an important potential therapeutic target [62–72]. These findings indicate there are CTAs that can directly modulate transcription, and we postulate that TEX19 could function in a similar fashion controlling cellular transcript levels, either at the transcriptional and/or post-transcriptional levels. As for mouse Tex19.1, TEX19 in cancer cells can operate on a small sub-set of protein coding and/or TE transcripts, although the latter may be indirect.

### TEX19 is both cytoplasmic and nuclear in cancer cells and testis cells

Consistent with a direct role in transcriptional regulation, we demonstrate that human TEX19 can locate to the nucleus, although there is no apparent nuclear localization/export signals on TEX19, which might suggest nuclear localization in response to cellular status is regulated by interacting partners. Murine Tex19.1 interacts with the E3 ubiquitin ligase Ubr2 which contains a nuclear localization signal, so a role for a UBR2-TEX19 interaction in human cancers would be worthy of further investigation [73]. TEX19 nuclear localisation appears to be linked to cell density and may be related to proliferative state, with nuclear TEX19 being associated with proliferation. Whilst murine Tex19.1 is predominantly localized to the cytoplasm, nuclear Tex19.1 can be observed in placental tissues indicating commonalities between murine and human proteins [33, 34]. Additionally, we note nuclear foci of TEX19 in testis, germ cell tumour line NTERA2 and LMB treated nuclei; the functional relevance, if any, is unknown although it is a noteworthy observation.

Recently, immunohistochemistry studies indicated that human TEX19 was localized to Sertoli cells [31]. Our immunofluorescence analysis of co-staining with the Sertoli cell marker vimentin did not show direct co-localisation of the high intensity TEX19 staining regions (although there appears to be a low intensity TEX19 staining throughout the basal region, including Sertoli cells), rather a close association, suggesting that TEX19 may not be exclusively Sertoli cell specific. Furthermore, IHC staining of normal testis appears to show nuclear localization/speckling in some of the large nuclei [31], consistent with our observation. Zhong and co-workers [31] extended their analysis to demonstrate that TEX19 was present in bladder cancer samples. Whilst most of the TEX19 they observed in these samples appeared to be cytoplasmic, there are clearly cells within the tumours that exhibit some nuclear staining with the anti-TEX19 antibodies employed [31].

### TEX19 expression influences clinical outcomes

That *TEX19* expression helps drive proliferative potential of cancer cells suggests that it might influence disease progression / outcome in patients. Our analysis of overall survival indicates that for a number of cancers, including breast cancer and renal cancers, that there is a significant correlation between higher *TEX19* expression and poor prognosis, supporting a potential functional association. These analyses might be an underestimation of the influence of *TEX19* expression as our analysis was based on splitting cancer patient cohorts based on median *TEX19* expression. Use of the median split means that the two populations being compared both have high numbers of *TEX19* expressing cancers; if *TEX19* expression alone (irrespective of the levels) is sufficient to drive cancers, then in many of our analyses a split based on the median would not detect a correlative link between *TEX19* expression and a poor prognosis. Ideally, we would have split all cohorts into those expressing *TEX19* and those not. This approach, however, was untenable as many data sets had an imbalance of negative *vs*. positive *TEX19* expressing cancers, negating statistical analysis. Moreover, this is further complicated by the difficulties of ascribing tumour biopsies as having no expression given the sensitivity/depth of modern deep RNA sequencing technologies, which can identify low abundance transcripts for almost all annotated genes [74]. Despite these limitations, the analysis of breast cancers and renal cancers clearly indicate a correlative link between high *TEX19* expression and poor prognosis, consistent with a functional role for TEX19 as an oncogenic proliferative driver.

Surprisingly, we observed the converse relationship for gliomas where higher *TEX19* expression is linked to a better prognosis, suggesting it has favorable activity in neuronal cells. This inverse influence on disease outcome has also been observed for T-box transcription factors, which both positively/negatively regulate gene expression during embryonic development [75]. For example, as for TEX19, TBX3 appears to have tumour suppressing activity in glioblastomas [76] but oncogenic activity in a number of solid tumours (for examples, see [77–79]; for review, see [75]). This commonality between T-box transcription factors and TEX19 could infer a functional link in the distinct regulation of developmental/proliferative genes in distinct cancer types. The factors that set neuronal tumours apart from other tumour types are likely to be multifold, however, it is noteworthy that *LINE-1* retrotransposition is active in somatic neuronal cells [80]; it is not unreasonable to postulate that TEX19 production in neuronal malignancies could limit *LINE-1* transposition events and thus limit the evolutionary capacity of the diseased genome and therefore the aggressiveness of the tumour.

## Conclusion

Here we have identified human TEX19 as a driver of cancer cell proliferative potential. Importantly, we demonstrate that depletion of TEX19 may result in S-phase defects, however, the exact functional role of TEX19 remains unclear. Evidence is starting to emerge to indicate that TEX19 is a transcriptional/transcript regulator that has a degree of plasticity, which may be modulated dependent upon the cell/tissue requirements. Whatever the exact role, it is clear that *TEX19* expression influences cancer prognosis and should be considered as a highly specific target for the development of novel anti-cancer therapeutic agents.

## Additional files

Additional file 1: Table S1. Human cancer cell lines used in this study. (DOCX 13 kb)

Additional file 2: Table S2. PCR primers used in the primary study. (DOCX 12 kb)

Additional file 3: Table S3. RT-qPCR analysis of 52 transcripts in TEX19 depleted cancer cell lines. The 52 transcripts were determined as the top hits (*P* < 0.01) from the RNA-seq data for transcript changes in SW480 (see Additional file 8; Figure S5). (DOCX 21 kb)

Additional file 4: Figure S1. *TEX19* is required for proliferation in cancer cells. a siRNA depletion of *TEX19* mRNA in SW480 using siRNA B results in loss of proliferative potential (line plot * ≤ 0.01). RT-qPCR analysis of *TEX19* mRNA levels at day 8 is given (bar chart; * ≤ 0.05; *** ≤ 0.001). Western blot analysis showing TEX19 depletion at 8 days is given (right). b siRNA depletion of *TEX19* mRNA in HCT116 using siRNA A results in loss of proliferative potential (line plot * ≤ 0.01). RT-qPCR analysis of *TEX19* mRNA levels at day 7 is given (bar chart; * ≤ 0.05; *** ≤ 0.001). Western blot analysis of TEX19 levels were not taken for this experiment (ND). c siRNA depletion of *TEX19* mRNA in H460 using siRNA A results in loss of proliferative potential (line plot * ≤ 0.01). RT-qPCR analysis of *TEX19* mRNA levels at day 8 is given (bar chart; * ≤ 0.05; *** ≤ 0.001). Western blot analysis showing TEX19 depletion at 8 days is given (right). d siRNA depletion of *TEX19* mRNA in NTERA2 using siRNA B results in loss of proliferative potential (line plot * ≤ 0.01). RT-qPCR analysis of *TEX19* mRNA levels at day 8 is given (bar chart; * ≤ 0.05; *** ≤ 0.001). Western blot analysis showing TEX19 depletion at 8 days is given (right). e Western blots showing siRNA A treatment results in depletion of TEX19 protein in SW480 cells. (PPTX 285 kb)

Additional file 5: Figure S2. *TEX19* is required for cancer progenitor/stem-like cell self-renewal. Sphere derived SW480 and NTERA2 cells were subjected to the extreme limiting dilution assay with siRNA depletion of TEX19. SW480 cells were treated with siRNA B and NTERA2 cells were treated with siRNA A. For both cell types there is a statistically significant difference between the *TEX19* specific siRNA and the control siRNA indicating a need for TEX19 for self-renewal (* ≤ 0.01). (PPTX 86 kb)

Additional file 6: Figure S3. Over expression of *TEX19* does not alter the proliferative potential of SW480 cancer cells. *TEX19* was introduced into SW480 cells under a DOX inducible promoter. Cell treated with DOX induced *TEX19* expression (RT-qPCR at 8 days shown in the right hand bar graph) do not have increased or reduced proliferation (left hand plot). (PPTX 13327 kb)

Additional file 7: Figure S4. Induction of a *TEX19* specific shRNA reduces proliferation of HCT116 cells. Left: A *TEX19* specific DOX inducible shRNA was integrated into HCT116 cells. Treatment with DOX results in a significant reduction in HCT116 proliferative capability (* ≤ 0.05). Right: RT-qPCR showing levels of TEX19 mRNA depletion. (PPTX 77 kb)

Additional file 8: Figure S5. TEX19 regulates 80 protein coding gene transcripts in cancer cells: a Heat map showing the pattern of changes in protein coding transcripts in SW480 cells depleted for *TEX19* mRNA. b List showing all the significant (*P* ≤ 0.05) log_2_ fold changes of protein coding transcripts in SW480 following depletion of *TEX19* mRNA. Red bars indicate a reduction in transcripts; blue bar indicates an increase in transcripts. *TEX19* is indicated in bold. ‘Inf’ represents infinite (positive Inf values indicate that genes were switched on from a previously undetectable state, whereas negative Inf values indicate that a given gene is switched to a state where no transcripts are detectable following siRNA treatment, but were prior to treatment). (PPTX 170 kb)

Additional file 9: Table S4. Table of cancer data sets analyzed. (DOCX 14 kb)

Additional file 10: Figure S6. Kaplan-Meier plots for renal cancer and glioma. a Kidney renal clear cell carcinoma (KIRC) has a reduced overall survival when there is high *TEX19* expression. Populations are divided by median *TEX19* expression (red = high; grey = low). Dashed lines are 95% confidence intervals. b Kidney renal cell carcinoma (KIRP) has a reduced overall survival when there is high *TEX19* expression. Populations are divided by median *TEX19* expression (red = high; grey = low). Dashed lines are 95% confidence intervals. c There is a marginal, but significantly better overall survival for lower grade glioma (LGG) patients with high levels of *TEX19* expression. Populations are divided by median *TEX19* expression (red = high; grey = low). Dashed lines are 95% confidence intervals. (PPTX 95 kb)

Additional file 11: Figure S7. Kaplan-Meier plots for lung and cervical cancer split by the highest 25% (red) *vs*. lower 75% (grey) for *TEX19* expression (excluding RNA-seq sets without full clinical data). a Lung adenocarcinoma (LUAD) has reduced overall survival when there is high *TEX19* expression. Dashed lines are 95% confidence intervals. b Cervical squamous cell carcinoma and endocervical adenocarcinoma (CESC) have reduced overall survival when there is high *TEX19* expression. Dashed lines are 95% confidence intervals. (PPTX 44 kb)

## Abbreviations

ATCC: American type culture collection
CT: Cancer/testis
CTA: Cancer/testis antigen
dox: Doxycycline
ECACC: European Collection of Authenticated Cell Cultures
ELDA: Extreme limiting dilution assay
ESC: Embryonic stem cell
FBS: Fetal bovine serum
HERV: Human endogenous retro virus
LMB: Leptomycin B
LTR: Long terminal repeat
TCGA: The Cancer Genome Atlas
TE: Transposable element
