# Supplementary material for: Human germ/stem cell-specific gene TEX19 influences cancer cell proliferation and cancer prognosis
Source: Mol Cancer. 2017 Apr 26;16:84. doi: 10.1186/s12943-017-0653-4 (PMC5406905; doi:10.1186/s12943-017-0653-4)
Supplement: Supplementary file 1 — Human cancer cell lines used in this study. (DOCX 13 kb) [file 12943_2017_653_MOESM1_ESM.docx]

**Table S1.** Cell lines employed in this study

| **Cell line** | **Origin** | **Phenotype** | **Source** |
| --- | --- | --- | --- |
| SW480 | Duke’s type B colorectal adenocarcinoma | Adherent with epithelial morphology | ECACC, Porton Down, UK  (Cat. No.:87092801) |
|  |  |  |  |
| HCT116 | Colorectal carcinoma | Adherent with epithelial morphology | ECACC, Porton Down, UK  (Cat. No.:91091005) |
|  |  |  |  |
| H460 | Large cell lung carcinoma | Adherent with epithelial morphology | ATCC, Teddington, UK  (ATCC No: HTB-177) |
|  |  |  |  |
| A2780 | Ovarian carcinoma | Adherent with epithelial morphology | ECACC, Porton Down, UK  (Cat. No.:93112519) |
|  |  |  |  |
|  | Derived from malignant effusion from the peritoneal ascites of a patient with well differentiated serous ovarian carcinoma | Adherent with epithelial morphology | ECACC, Porton Down, UK  (Cat. No.:10032311) |
|  |  |  |  |
| NTERA2 | Lung metastasis of pluripotent embryonal carcinoma | Adherent with epithelia-like morphology and are capable of differentiation and phenotype changes | Gift. Prof. P.Andrews (University of Sheffield, UK) |
|  |  |  |  |
| HeLa S3 | Cervical adenocarcinoma | Adherent with epithelial morphology | ATCC, Teddington, UK  (ATCC No: CCL-2) |
|  |  |  |  |
| LoVo | Colon adenocarcinoma | Adherent with epithelial morphology | ECACC, Porton Down, UK  (Cat. No.:87060101) |
|  |  |  |  |
| SW626 | Ovarian metastasis of colon adenocarcinoma | Adherent with epithelial morphology | ECACC, Porton Down, UK  (Cat. No.:91091203) |
|  |  |  |  |
| T84 | Lung metastasis of colon carcinoma | Adherent with epithelial morphology | ECACC, Porton Down, UK  (Cat. No.:88021101) |
