# Supplementary material for: Human germ/stem cell-specific gene TEX19 influences cancer cell proliferation and cancer prognosis
Source: Mol Cancer. 2017 Apr 26;16:84. doi: 10.1186/s12943-017-0653-4 (PMC5406905; doi:10.1186/s12943-017-0653-4)
Supplement: Supplementary file 2 — PCR primers used in the primary study. (DOCX 12 kb) [file 12943_2017_653_MOESM2_ESM.docx]

**Table S2.** RT-qPCR primer sequences for HERV expression analysis.

| **HERV gene** | **Primer designation** | **Primer sequence (5' – 3')** |
| --- | --- | --- |
|  |  |  |
| HERVK 107 | ERVK107F  ERVK107R | GAGAGCCTCCCACAGTTGAG  TTTGCCAGAATCTCCCAATC |
| HERVK gag | ERVK Gag F  ERVK Gag R | CCCGACATTTGTCTTGGTCT  CCTGGGGAATCCTTCTCTTC |
| HERVK pro | ERVK Pro F  ERVK Pro R | TGTTCCTCAGGGTTTTCAGG  CCCTGGAAGCAGAGAGACTG |
| HERVK HML2 rec | ERVK Rec F  ERVK Rec R | ATGAACCCATCGGAGATGCA AACAGAATCTCAAGGCAGAAGA |
