# Supplementary material for: Human germ/stem cell-specific gene TEX19 influences cancer cell proliferation and cancer prognosis
Source: Mol Cancer. 2017 Apr 26;16:84. doi: 10.1186/s12943-017-0653-4 (PMC5406905; doi:10.1186/s12943-017-0653-4)
Supplement: Supplementary file 3 — RT-qPCR analysis of 52 transcripts in TEX19 depleted cancer cell lines. The 52 transcripts were determined as the top hits (P < 0.01) from the RNA-seq data for transcript changes in SW480 (see Additional file 8; Figure S5). (DOCX 21 kb) [file 12943_2017_653_MOESM3_ESM.docx]

**Table S3.** RT-qPCR analysis of 52 transcripts in TEX19 depleted cancer cell lines.

| **Gene** | **RNA-seq**  **Log2-fold change (SW480)** | **Independent**  **RT-qPCR**  **SW480** | **RT-qPCR**  **H460** | **RT-qPCR**  **A2780** | **RT-qPCR**  **NTERA2** | **RT-qPCR**  **PEO14** | **PCR primer sequences**  **or source company reference**  **(F-forward/R-reverse)** |  |
| --- | --- | --- | --- | --- | --- | --- | --- | --- |
|  |  |  |  |  |  |  |  |  |
| *MIPEP* | -1.13 | NC | **🡑** | NC | **🡓** | n.d. | F 5'-CTAGCACCATACCCCACGAG-3'  R 5'-TCAACCACAGACATTCTCAAGG-3' | |
| *CYFIP1* | -1.54 | NC | **🡓** | n.d. | NC | **🡓** | F 5'-AGGTCCACGTTGGACAGC-3'  R 5'-GCCAAGGAAACTCCCAGG-3' | |
| *GPR137B* | -2.33 | NC | **🡓** | n.d. | **🡓** | n.d. | F 5'-GGGCAACCGGTATTTGAGTA-3'  R 5'-ACCCTCACGCTGATGAACTT-3' | |
| *PGM1* | -1.43 | n.d. | **🡓** | NC | **🡓** | n.d. | F 5'-GAGGATTCCATTCTGTCCGA-3'  R 5'-CCGGTTCTACATGAAGGAGG-3' | |
| *ATG16L1* | -1.22 | **🡑** | NC | n.d. | NC | n.d. | F 5'-GCATTGTCCAGCAGGAACTT-3'  R 5'-AAATGATTTTGCAAGCCGAA-3' | |
| *GINS1* | -1.14 | **🡑** | **🡓** | **🡓** | n.d. | **🡓** | F 5'-AAGAAATCGACGCTGCACTG-3'  R 5'-TCAGCAGCCATGTGAAATCG-3' | |
| *DTWD1* | -1.16 | NC | NC | n.d. | NC | n.d. | F 5'-CGCCAAAAGCAAGTTTTTCT-3'  R 5'-CACCTGGAACCAAACAAACA-3' | |
| *CCNE1* | -1.75 | NC | NC | n.d. | NC | n.d. | F 5'-CCACACCTGACAAAGAAGATGATGAC-3'  R 5'-GAGCCTCTGGATGGTGCAATAAT-3' | |
| *GAR1* | -1.59 | **🡑** | **🡓** | **🡓** | NC | **🡓** | F 5'-CACCTGGAGGTCGAGGTAAA-3'  R 5'-TTGTCAGAAAACATGAAGGCT-3' | |
| *FHL2* | -1.27 | **🡑** | NC | n.d. | NC | n.d. | F 5'-GTGCCGGTCCTTGTAAGACA-3'  R 5'-GGTGTGCTTTGAGACCCTGT-3' | |
| *MRS2* | -1.25 | **🡑** | NC | n.d. | NC | n.d. | F 5'-GGCCACTTCCCATGAACATA-3'  R 5'-AATGGGAGTTGCTTTTGGAA-3' | |
| *NAPB* | -1.19 | NC | NC | NC | **🡓** | n.d. | F 5'-CCTGCAGCACTCCAATTTTT-3'  R 5'-CTTCCTCCGAGGGCTGTTT-3' | |
| *E2F5* | -1.12 | **🡑** | NC | **🡓** | n.d. | **🡑** | F 5'-CCTGTTCCCCCACCTGATG-3'  R 5'-TTTCTGTGGAGTCACTGGAGTCA-3' | |
| *CTSL2* | -1.21 | NC | NC | **🡓** | NC | **🡑** | F 5'-TTACATAGCCATTCGAGCCC-3'  R 5'-TACGGCTTTGAAGGAGCAAA-3' | |
| *MDC1* | -1.69 | **🡑** | **🡓** | **🡓** | NC | **🡑** | F 5'-AATGGCTGTGTAGCCAGGAC-3'  R 5'-CTTCATGTTGACTCCACCCC-3' | |
| *FAM117B* | -1.36 | **🡑** | **🡓** | n.d. | NC | n.d. | F 5'-TACTGGAGGGGCTTGAAGAA-3'  R 5'-AGGTCTCCGGAGCAGAGC-3' | |
| *ANKRD52** | -1.31 | n.d. | n.d. | n.d. | n.d. | n.d. | Primers failed | |
| *GALNT2* | -1.19 | **🡑** | NC | **🡑** | **🡓** | n.d. | F 5'-CCATTGCTGTGATGAAGGTC-3'  R 5'-CTGGGCATCGCCTACTACAT-3' | |
| *MTA2* | -1.47 | NC | **🡓** | **🡓** | NC | **🡓** | F 5'-CCGACGGCCTTATGCTCCT-3'  R 5'-CTGGGCCACCAGATCTTTGAC-3' | |
| *DIXDC1* | -1.21 | **🡑** | NC | **🡓** | NC | **🡓** | F 5'-ACAGGTGCTGCTGACAGTTG-3'  R 5'-GGGTCAAGTCACCCAGAACT-3' | |
| *RPIA* | -1.51 | **🡓** | NC | NC | **🡓** | n.d. | F 5'-CGATCCAGATCACTGAGGGT-3'  R 5'-GCTGAAAGGGTGAAGCAAGA-3' | |
| *LONRF1* | -1.06 | **🡑** | NC | n.d. | NC | n.d. | F 5'-TCAGACAGTTCATCAGGCAGA-3'  R 5'-GCACCATATTGTCCTCTTTGC-3' | |
| *ELK4* | -1.02 | **🡑** | **🡓** | NC | **🡓** | n.d. | F 5'-AACTGCCACAGGGTGATAGC-3'  R 5'-AGTTTCCAGCGTGAGGAGG-3' | |
| *ATP5C1* | -1.48 | NC | **🡑** | NC | **🡓** | n.d. | F 5'-TGATGGACTTTAGTCTCCTGGTG-3'  R 5'-GGCTGTGGCTACCATGTTCT-3' | |
| *ZBTB5* | -1.09 | NC | **🡓** | NC | **🡓** | n.d. | F 5'-CAAAGTGACCAGGAAAATCCA-3'  R 5'-CACCTCCTCTGACACTGCC-3' | |
| *FAM98B* | -1.21 | **🡑** | **🡓** | n.d. | NC | n.d. | F 5'-AGATAATCCACCCTCTGCCG-3'  R 5'-GAGACGTGCTGGACACACTG-3' | |
| *MRPS23* | -1.02 | NC | NC | **🡓** | NC | n.d. | F 5'-TCTCCACAAACCGTTGACAG-3'  R 5'-CACGAGGATCGGATTAGAGC-3' | |
| *TSN* | -1.29 | NC | **🡓** | **🡓** | NC | **🡓** | F 5'-ACACAACAAATGCTGCCAAG-3'  R 5'-TGAAGACCAAATTTCCTGCTG-3' | |
| *ZCCHC2* | -1.08 | **🡑** | **🡓** | n.d. | NC | n.d. | F 5'-GGGAGTCTGATGATATGGACTG-3'  R 5'-ACATCCTGACCTAGAGCCCA-3' | |
| *ZBTB18* | -1.32 | **🡑** | NC | n.d. | NC | n.d. | F 5'-TCGCTCAGACACTGTAGCAAA-3'  R 5'-CCAGCAGGACTCAGAGGAAA-3' | |
| *SAA2* | -1.02 | **🡓** | **🡓** | **🡓** | **🡑** | n.d. | F 5'-GTAGGCTCTCCACATGTCCC-3'  R 5'-TGGTTTTCTGCTCCTTGGTC-3' | |
| *CDHR2* | -1.48 | **🡑** | NC | n.d. | NC | n.d. | F 5'-TCCCATAGGTCAGAGGGTCA-3'  R 5'-CAGTGATCCTGCCTGAGGAC-3' | |
| *INPP4B* | -1.03 | NC | NC | n.d. | NC | n.d. | F 5'-TTCTGGTAGGACACTGGTTCG-3'  R 5'-CATTCCCATCTGAGTATCCCA-3' | |
| *RAD21L1* | -1.05 | - | - | - | - | - | QT02508401  Qiagen | |
| *MYB* | -1.39 | **🡓** | **🡓** | **🡓** | NC | **🡓** | F 5'-GCAGGTTCCCAGGTACTGCT-3'  R 5'-GCACCAGCATCAGAAGATGA-3' | |
| *ARHGAP9* | -1.44 | - | - | - | - | - | F 5'-GGCCACTCCTCTTTTCCTCT-3'  R 5'-ACAATCGGCTGAGAATGAGAA-3' | |
| *C4BPB* | -1.44 | NC | NC | - | NC | - | F 5'-TAGCCATGAACTGGATTCCC-3'  R 5'-TCGGAGCCAGTGTCTAGAGG-3' | |
| *TEX19* | -1.12 | **🡓** | **🡓** | **🡓** | **🡓** | **🡓** | F 5'-GTGCCCACATGAACAGAGAC-3'  R 5'-GGTTTCCTATCCAGGGATTTGG-3' | |
| *TNC* | 1.26 | **🡑** | NC | **🡑** | **🡑** | **🡑** | F 5'-CTGTTGGCAGGTGTCTTTCTT-3'  R 5'-GTGCCGGATGACTTTCTTGAG-3' | |
| *MYL9* | 1.31 | **🡓** | **🡓** | **🡓** | NC | **🡓** | F 5'-CTTGCTGGACATCTTGGCTT-3'  R5'-GGAGTCCAGACCCGACG-3' | |
| *PTBP2* | 1.32 | **🡑** | **🡓** | **🡓** | NC | **🡑** | F 5'-TCGTCAGATCCTCTCTTCACG-3'  R 5'-CTCGGTTCTTGTGAGCGAA-3' | |
| *VIL1* | 1.22 | **🡓** | NC | **🡑** | NC | **🡓** | F 5'-ACACAGGTGGAGGTGCAGAAT-3'  R 5'-GGTTGGTCGCTGTCCACTTC-3' | |
| *TAGLN* | 1.47 | **🡓** | **🡑** | **🡓** | **🡓** | n.d. | F 5'-CTCATGCCATAGGAAGGACC-3'  R 5'-GGCCAAGGCTCTACTGTCTG-3' | |
| *NOS3* | 1.03 | NC | NC | NC | **🡓** | n.d. | F 5'-GACATCTCCATCAGGGCAG-3'  R 5'-TGAGTATGACGTGGTGTCCC-3' | |
| *ZNF367* | 1.18 | **🡑** | NC | **🡑** | **🡓** | n.d. | F 5'-AATCGCGGACAGTATCTGCT-3'  R 5'-GTGAGGACGAGGAGGAAGC-3' | |
| *PIWIL1* | 1.26 | **🡑** | - | - | NC | n.d. | QT00064638  Qiagen | |
| *CD22* | 1.08 | **🡑** | NC | NC | **🡓** | n.d. | F 5'-CTGGAGGGAGCTGGATATGA-3'  R 5'-GCTGAGGATGGAGTCCAAGA-3' | |
| *SLC17A7* | 1.19 | **🡑** | **🡑** | **🡑** | **🡓** | n.d. | F 5'-ACTCAGCTCCAGCGTCTCC-3'  R 5'-GAGTTTCGGAAGCTAGCGG-3' | |
| *ASB2* | 1.31 | **🡑** | NC | **🡓** | **🡓** | n.d. | F 5'-CAGGTGCAGCGGACTGA-3'  R 5'-AACAAGACCAACAAGGACGG-3' | |
| *C2orf78** | 2.36 | n.d. | n.d. | n.d. | n.d. | n.d. | Primers failed | |
| *CPS1* | 1.40 | **🡑** | NC | **🡑** | **🡓** | n.d. | F 5'-CCCAAGGCATTTTGAAATCT-3'  R 5'-GGAAAAGACACTGAAAGGGCT-3' | |
| *CALB2* | 1.19 | **🡑** | **🡑** | NC | NC | n.d. | F 5'-GACATCATGCCAGAGCCTTT-3'  R 5'-TATGGAAGCACTTTGACGCA-3' | |

Note: Arrows (**🡑** & **🡓**) indicate direction of transcript level change associated with a P value of < 0.01.

- = no transcript measurable.

n.d. = note determined due to failure to detect transcripts using RT-qPCR primer set.

NC = no change upon TEX19 siRNA treatment.

Green = up regulated; Red = down regulated; grey = no change (NC); white = not determined or no detectable transcripts.

*PCR primer sets failed – no data obtained/PCR primers recorded.
