# Supplementary material for: Human germ/stem cell-specific gene TEX19 influences cancer cell proliferation and cancer prognosis
Source: Mol Cancer. 2017 Apr 26;16:84. doi: 10.1186/s12943-017-0653-4 (PMC5406905; doi:10.1186/s12943-017-0653-4)
Supplement: Supplementary file 4 — TEX19 is required for proliferation in cancer cells. a siRNA depletion of TEX19 mRNA in SW480 using siRNA B results in loss of proliferative potential (line plot * ≤ 0.01). RT-qPCR analysis of TEX19 mRNA levels at day 8 is given (bar chart; * ≤ 0.05; *** ≤ 0.001). Western blot analysis showing TEX19 depletion at 8 days is given (right). b siRNA depletion of TEX19 mRNA in HCT116 using siRNA A results in loss of proliferative potential (line plot * ≤ 0.01). RT-qPCR analysis of TEX19 mRNA levels at day 7 is given (bar chart; * ≤ 0.05; *** ≤ 0.001). Western blot analysis of TEX19 levels were not taken for this experiment (ND). c siRNA depletion of TEX19 mRNA in H460 using siRNA A results in loss of proliferative potential (line plot * ≤ 0.01). RT-qPCR analysis of TEX19 mRNA levels at day 8 is given (bar chart; * ≤ 0.05; *** ≤ 0.001). Western blot analysis showing TEX19 depletion at 8 days is given (right). d siRNA depletion of TEX19 mRNA in NTERA2 using siRNA B results in loss of proliferative potential (line plot * ≤ 0.01). RT-qPCR analysis of TEX19 mRNA levels at day 8 is given (bar chart; * ≤ 0.05; *** ≤ 0.001). Western blot analysis showing TEX19 depletion at 8 days is given (right). e Western blots showing siRNA A treatment results in depletion of TEX19 protein in SW480 cells. (PPTX 285 kb) [file 12943_2017_653_MOESM4_ESM.pptx]

## Slide 1
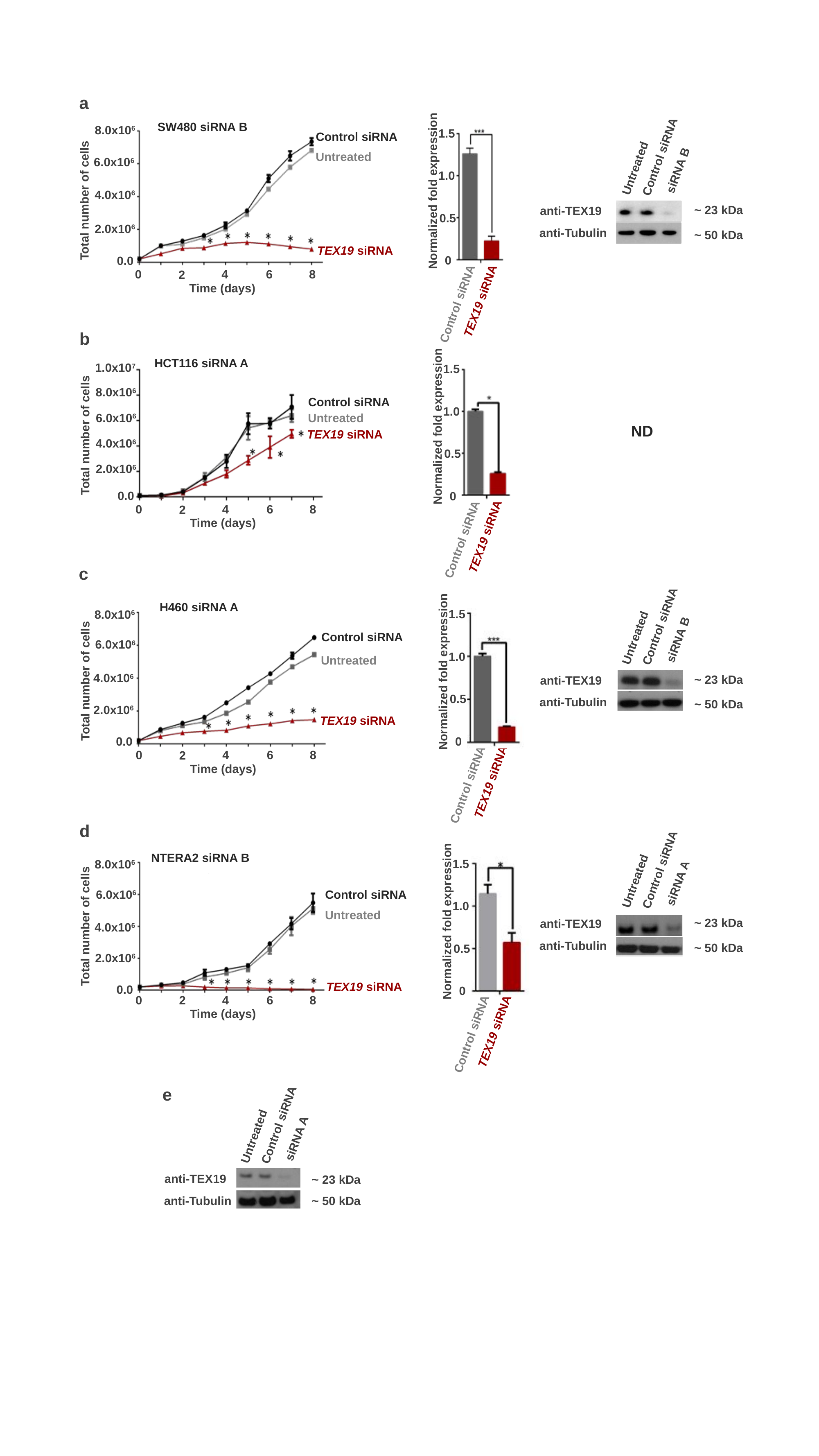

a
1.5
1.0
Normalized fold expression
0.5
0
TEX19 siRNA
Control siRNA
Control siRNA
Untreated
siRNA B
~ 23 kDa
anti-TEX19
anti-Tubulin
~ 50 kDa
SW480 siRNA B
8.0x10^6^
Control siRNA
Untreated
6.0x10^6^
4.0x10^6^
Total number of cells
2.0x10^6^
TEX19 siRNA
0.0
0
4
6
8
2
Time (days)
b
1.5
1.0
Normalized fold expression
0.5
0
TEX19 siRNA
Control siRNA
HCT116 siRNA A
1.0x10^7^
8.0x10^6^
Control siRNA
6.0x10^6^
Untreated
TEX19 siRNA
Total number of cells
4.0x10^6^
2.0x10^6^
0.0
0
4
6
8
2
Time (days)
ND
c
Control siRNA
Untreated
siRNA B
~ 23 kDa
anti-TEX19
anti-Tubulin
~ 50 kDa
1.5
1.0
Normalized fold expression
0.5
0
TEX19 siRNA
Control siRNA
H460 siRNA A
8.0x10^6^
Control siRNA
6.0x10^6^
Untreated
4.0x10^6^
Total number of cells
2.0x10^6^
TEX19 siRNA
0.0
0
4
6
8
2
Time (days)
d
Control siRNA
Untreated
siRNA A
~ 23 kDa
anti-TEX19
anti-Tubulin
~ 50 kDa
1.5
1.0
Normalized fold expression
0.5
0
TEX19 siRNA
Control siRNA
NTERA2 siRNA B
8.0x10^6^
6.0x10^6^
Control siRNA
Untreated
Total number of cells
4.0x10^6^
2.0x10^6^
TEX19 siRNA
0.0
0
4
6
8
2
Time (days)
Control siRNA
Untreated
siRNA A
anti-TEX19
~ 23 kDa
anti-Tubulin
~ 50 kDa
e
