# Supplementary material for: Human germ/stem cell-specific gene TEX19 influences cancer cell proliferation and cancer prognosis
Source: Mol Cancer. 2017 Apr 26;16:84. doi: 10.1186/s12943-017-0653-4 (PMC5406905; doi:10.1186/s12943-017-0653-4)
Supplement: Supplementary file 5 — TEX19 is required for cancer progenitor/stem-like cell self-renewal. Sphere derived SW480 and NTERA2 cells were subjected to the extreme limiting dilution assay with siRNA depletion of TEX19. SW480 cells were treated with siRNA B and NTERA2 cells were treated with siRNA A. For both cell types there is a statistically significant difference between the TEX19 specific siRNA and the control siRNA indicating a need for TEX19 for self-renewal (* ≤ 0.01). (PPTX 86 kb) [file 12943_2017_653_MOESM5_ESM.pptx]

## Slide 1
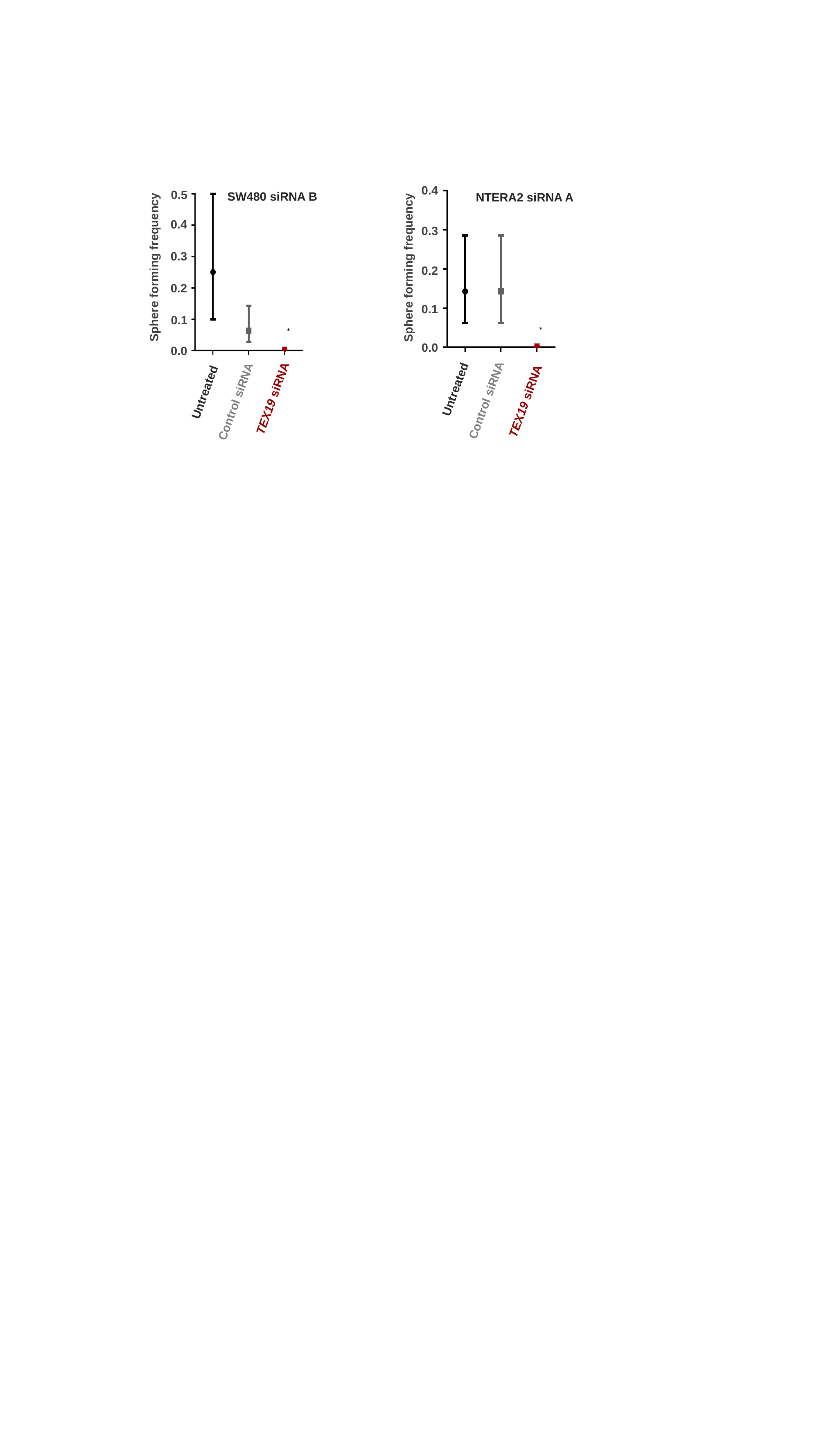

0.4
NTERA2 siRNA A
0.3
Sphere forming frequency
0.2
0.1
0.0
Untreated
Control siRNA
TEX19 siRNA
0.5
SW480 siRNA B
0.4
0.3
Sphere forming frequency
0.2
0.1
0.0
Untreated
TEX19 siRNA
Control siRNA
*
*
