# Supplementary figures and images for: Human germ/stem cell-specific gene TEX19 influences cancer cell proliferation and cancer prognosis
Source: Mol Cancer. 2017 Apr 26;16:84. doi: 10.1186/s12943-017-0653-4 (PMC5406905; doi:10.1186/s12943-017-0653-4)

## Slide 1
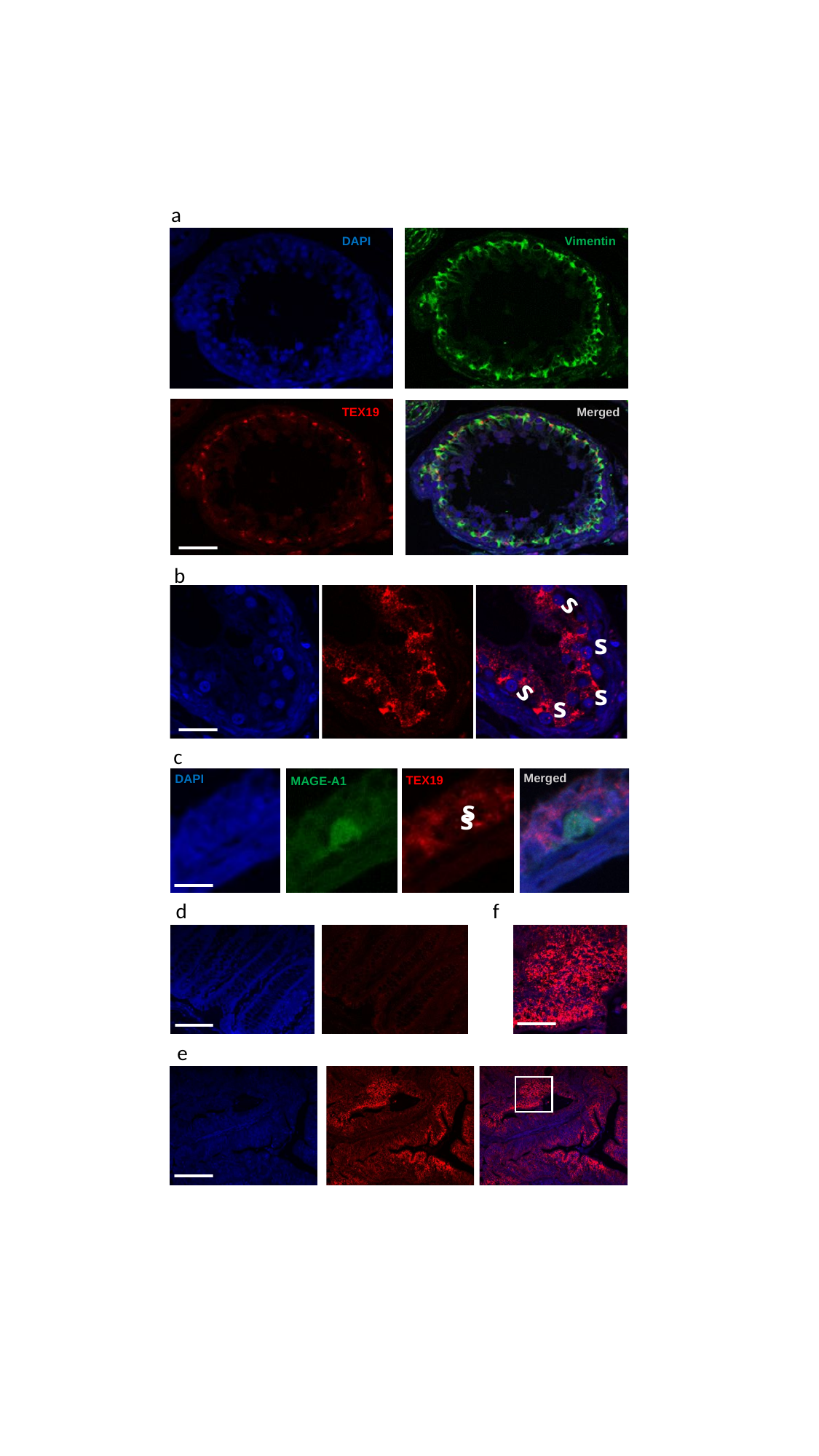

a
DAPI
Vimentin
TEX19
Merged
b
s
s
s
s
s
c
Merged
DAPI
TEX19
MAGE-A1
s
s
d
f
e

Supplement: Supplementary file 6 — Over expression of TEX19 does not alter the proliferative potential of SW480 cancer cells. TEX19 was introduced into SW480 cells under a DOX inducible promoter. Cell treated with DOX induced TEX19 expression (RT-qPCR at 8 days shown in the right hand bar graph) do not have increased or reduced proliferation (left hand plot). (PPTX 13327 kb) [file 12943_2017_653_MOESM6_ESM.pptx]

## Slide 1
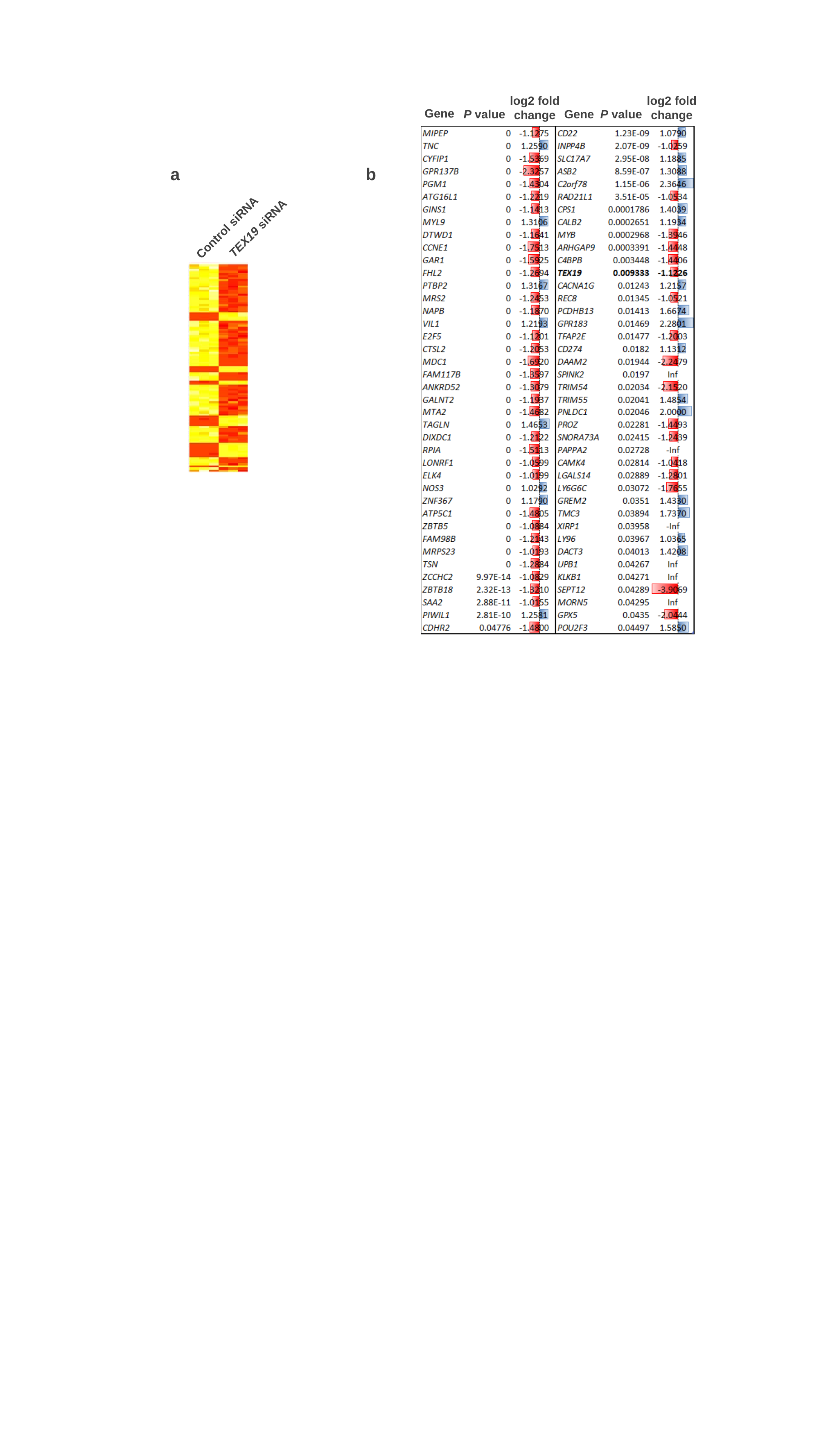

log2 fold
change
log2 fold
change
Gene
P value
P value
Gene
B
a
b
Control siRNA
TEX19 siRNA

Supplement: Supplementary file 8 — TEX19 regulates 80 protein coding gene transcripts in cancer cells: a Heat map showing the pattern of changes in protein coding transcripts in SW480 cells depleted for TEX19 mRNA. b List showing all the significant (P ≤ 0.05) log2 fold changes of protein coding transcripts in SW480 following depletion of TEX19 mRNA. Red bars indicate a reduction in transcripts; blue bar indicates an increase in transcripts. TEX19 is indicated in bold. ‘Inf’ represents infinite (positive Inf values indicate that genes were switched on from a previously undetectable state, whereas negative Inf values indicate that a given gene is switched to a state where no transcripts are detectable following siRNA treatment, but were prior to treatment). (PPTX 170 kb) [file 12943_2017_653_MOESM8_ESM.pptx]
