# Supplementary material for: Human germ/stem cell-specific gene TEX19 influences cancer cell proliferation and cancer prognosis
Source: Mol Cancer. 2017 Apr 26;16:84. doi: 10.1186/s12943-017-0653-4 (PMC5406905; doi:10.1186/s12943-017-0653-4)
Supplement: Supplementary file 7 — Induction of a TEX19 specific shRNA reduces proliferation of HCT116 cells. Left: A TEX19 specific DOX inducible shRNA was integrated into HCT116 cells. Treatment with DOX results in a significant reduction in HCT116 proliferative capability (* ≤ 0.05). Right: RT-qPCR showing levels of TEX19 mRNA depletion. (PPTX 77 kb) [file 12943_2017_653_MOESM7_ESM.pptx]

## Slide 1
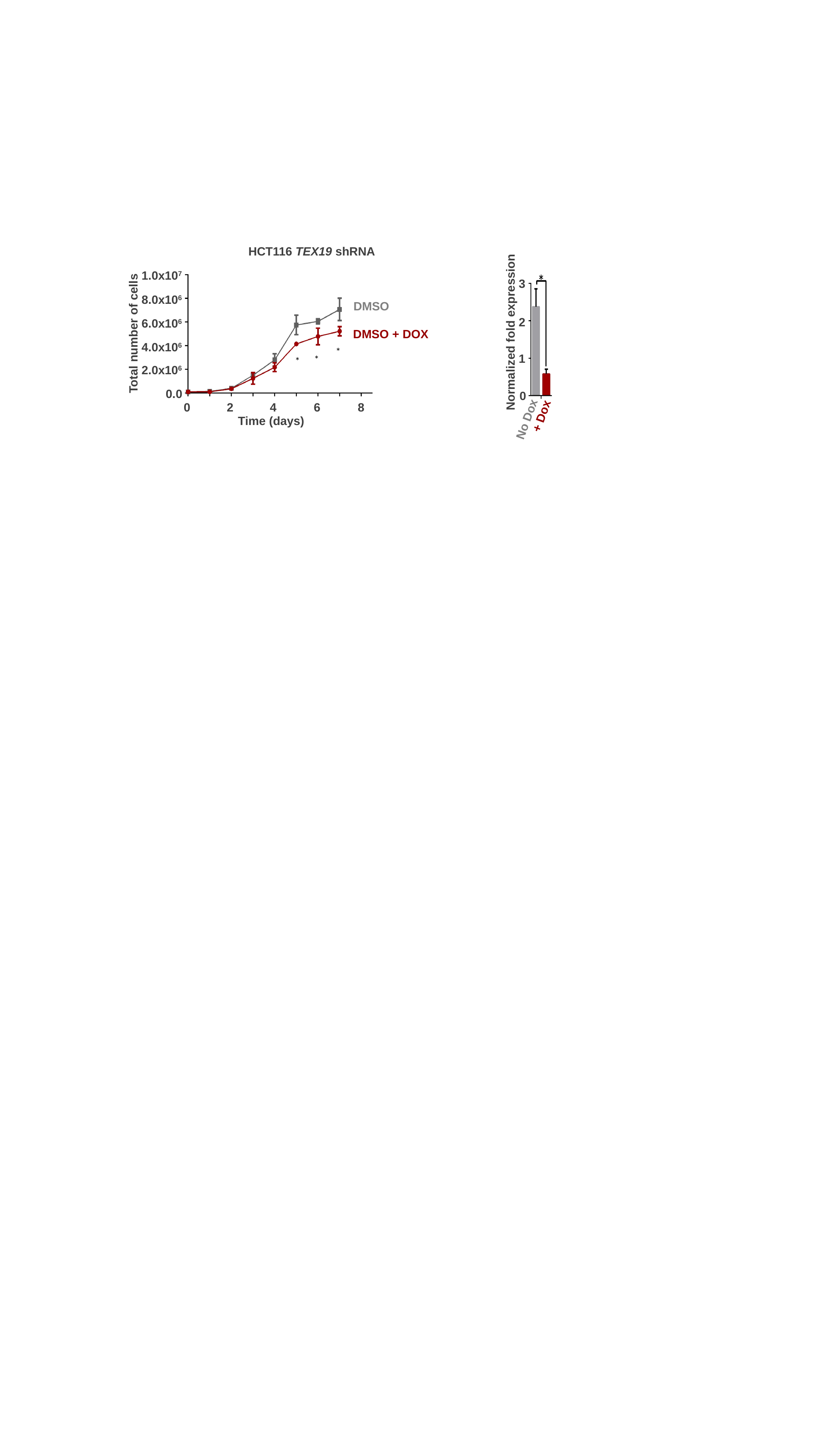

HCT116 TEX19 shRNA
1.0x10^7^
8.0x10^6^
DMSO
6.0x10^6^
Total number of cells
DMSO + DOX
4.0x10^6^
*
*
*
2.0x10^6^
0.0
0
2
4
6
8
Time (days)
3
2
Normalized fold expression
1
0
+ Dox
No Dox
