# Supplementary material for: Human germ/stem cell-specific gene TEX19 influences cancer cell proliferation and cancer prognosis
Source: Mol Cancer. 2017 Apr 26;16:84. doi: 10.1186/s12943-017-0653-4 (PMC5406905; doi:10.1186/s12943-017-0653-4)
Supplement: Supplementary file 9 — Table of cancer data sets analyzed. (DOCX 14 kb) [file 12943_2017_653_MOESM9_ESM.docx]

**Table S4.** Cancer cohorts used in the prognosis analysis.

| **Cohort** | **Cohort abbreviation** | **Number of data sets in cohort** |
| --- | --- | --- |
|  |  |  |
| Bladder urothelial carcinoma | BLCA | 408 |
| Breast invasive carcinoma | BRCA | 1093 |
| Cervical squamous cell carcinoma and endovervical adenocarcinoma | CESC | 304 |
| Colon adenocarcinoma | COAD | 191 |
| Colorectal adenocarcinoma | COADREAD | 263 |
|  |  |  |
| Esophageal carcinoma | ESCA | 184 |
|  |  |  |
| Glioblastoma multiforme | GBM | 152 |
|  |  |  |
| Glioma | GBMLGG | 667 |
|  |  |  |
| Head and neck squamous cell carcinoma | HNSC | 520 |
|  |  |  |
| Pan-kidney cohort | KIPAN | 889 |
|  |  |  |
| Kidney renal clear cell carcinoma | KIRC | 533 |
|  |  |  |
| Kidney renal papillary cell carcinoma | KIRP | 290 |
|  |  |  |
| Brain low grade glioma | LGG | 515 |
|  |  |  |
| Liver hepatocellular carcinoma | LIHC | 371 |
|  |  |  |
| Lung adenocarcinoma | LUAD | 515 |
|  |  |  |
| Lung squamous cell carcinoma | LUSC | 501 |
|  |  |  |
| Ovarian serous cystadenocarcinoma | OV | 303 |
|  |  |  |
| Pancreatic adenocarcinoma | PAAD | 178 |
|  |  |  |
| Pheochromocytoma and paraganglioma | PCPG | 179 |
|  |  |  |
| Prostate adenocarcinoma | PRAD | 497 |
|  |  |  |
| Sarcoma | SARC | 259 |
|  |  |  |
| Skin cutaneous melanoma | SKCM | 103 |
|  |  |  |
| Stomach adenocarcinoma | STAD | 415 |
|  |  |  |
| Stomach and esophageal carcinoma | STES | 599 |
|  |  |  |
| Testicular germ cell tumours | TGCT | 134 |
|  |  |  |
| Thymoma | THYM | 120 |
|  |  |  |
| Uterine corpus endometrial carcinoma | UCEC | 176 |

Note: Expression criteria for *TEX19* in data sets were set using the active gene designation of Hart and co-workers (Hart T, Komori HK, LaMere S, Podshivalova K, Salomon DR. Finding the active genes in deep RNA-seq gene expression studies. BMC Genomics. 2013;14:778.) This could mean that some biopsies are scored as not expressing *TEX19* when *TEX19* transcript levels are low. Given this, it is appropriate to split data sets by the median value scoring no expression in the lower half.

|  |
| --- |
|  |
|  |
