# Supplementary material for: Human germ/stem cell-specific gene TEX19 influences cancer cell proliferation and cancer prognosis
Source: Mol Cancer. 2017 Apr 26;16:84. doi: 10.1186/s12943-017-0653-4 (PMC5406905; doi:10.1186/s12943-017-0653-4)
Supplement: Supplementary file 10 — Kaplan-Meier plots for renal cancer and glioma. a Kidney renal clear cell carcinoma (KIRC) has a reduced overall survival when there is high TEX19 expression. Populations are divided by median TEX19 expression (red = high; grey = low). Dashed lines are 95% confidence intervals. b Kidney renal cell carcinoma (KIRP) has a reduced overall survival when there is high TEX19 expression. Populations are divided by median TEX19 expression (red = high; grey = low). Dashed lines are 95% confidence intervals. c There is a marginal, but significantly better overall survival for lower grade glioma (LGG) patients with high levels of TEX19 expression. Populations are divided by median TEX19 expression (red = high; grey = low). Dashed lines are 95% confidence intervals. (PPTX 95 kb) [file 12943_2017_653_MOESM10_ESM.pptx]

## Slide 1
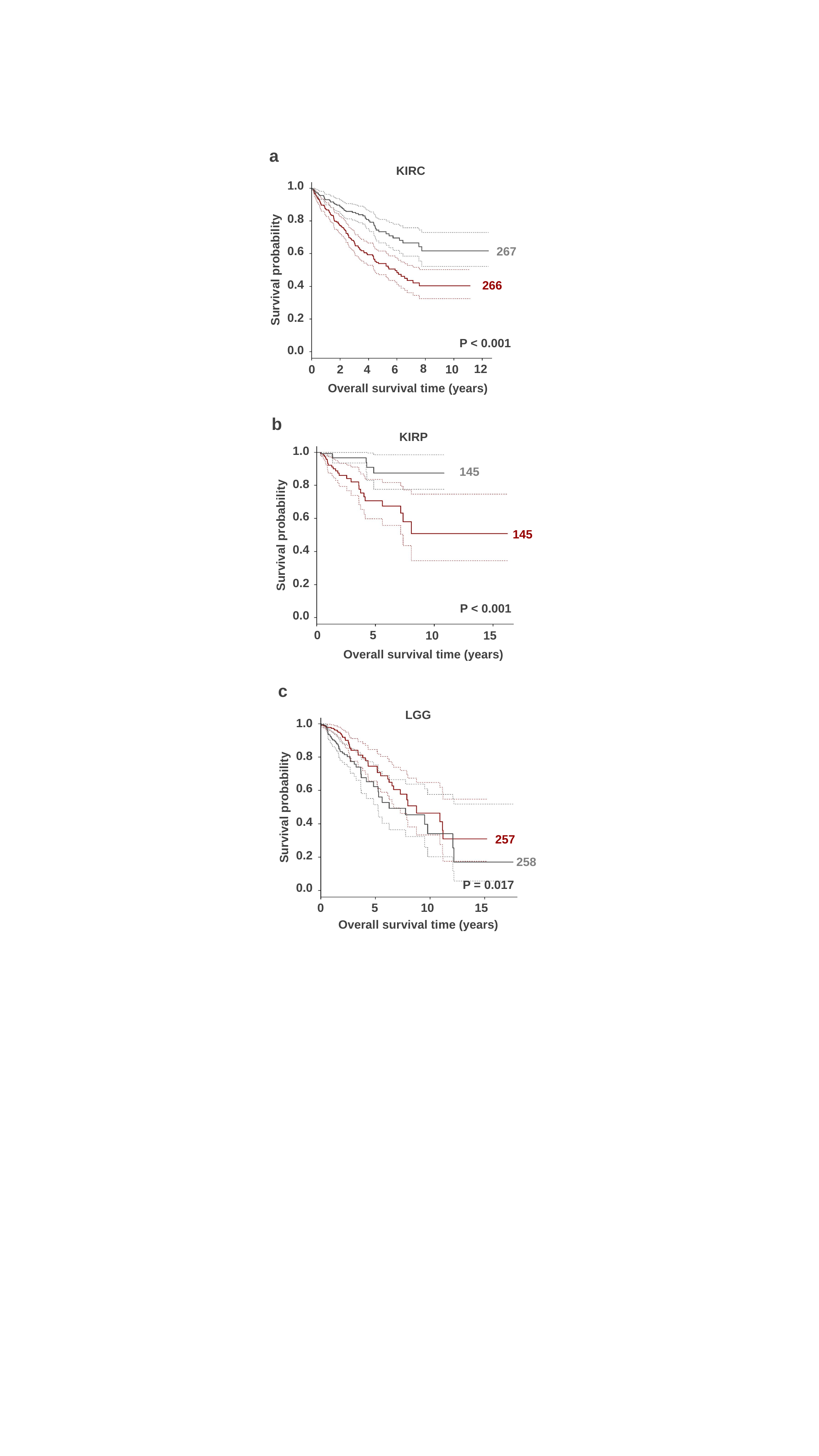

a
KIRC
1.0
0.8
0.6
267
Survival probability
0.4
266
0.2
P < 0.001
0.0
8
12
10
0
2
4
6
Overall survival time (years)
b
KIRP
1.0
145
0.8
0.6
145
Survival probability
0.4
0.2
P < 0.001
0.0
0
5
15
10
Overall survival time (years)
c
LGG
1.0
0.8
0.6
Survival probability
0.4
257
0.2
258
P = 0.017
0.0
0
5
10
15
Overall survival time (years)
