# Supplementary material for: Human germ/stem cell-specific gene TEX19 influences cancer cell proliferation and cancer prognosis
Source: Mol Cancer. 2017 Apr 26;16:84. doi: 10.1186/s12943-017-0653-4 (PMC5406905; doi:10.1186/s12943-017-0653-4)
Supplement: Supplementary file 11 — Kaplan-Meier plots for lung and cervical cancer split by the highest 25% (red) vs. lower 75% (grey) for TEX19 expression (excluding RNA-seq sets without full clinical data). a Lung adenocarcinoma (LUAD) has reduced overall survival when there is high TEX19 expression. Dashed lines are 95% confidence intervals. b Cervical squamous cell carcinoma and endocervical adenocarcinoma (CESC) have reduced overall survival when there is high TEX19 expression. Dashed lines are 95% confidence intervals. (PPTX 44 kb) [file 12943_2017_653_MOESM11_ESM.pptx]

## Slide 1
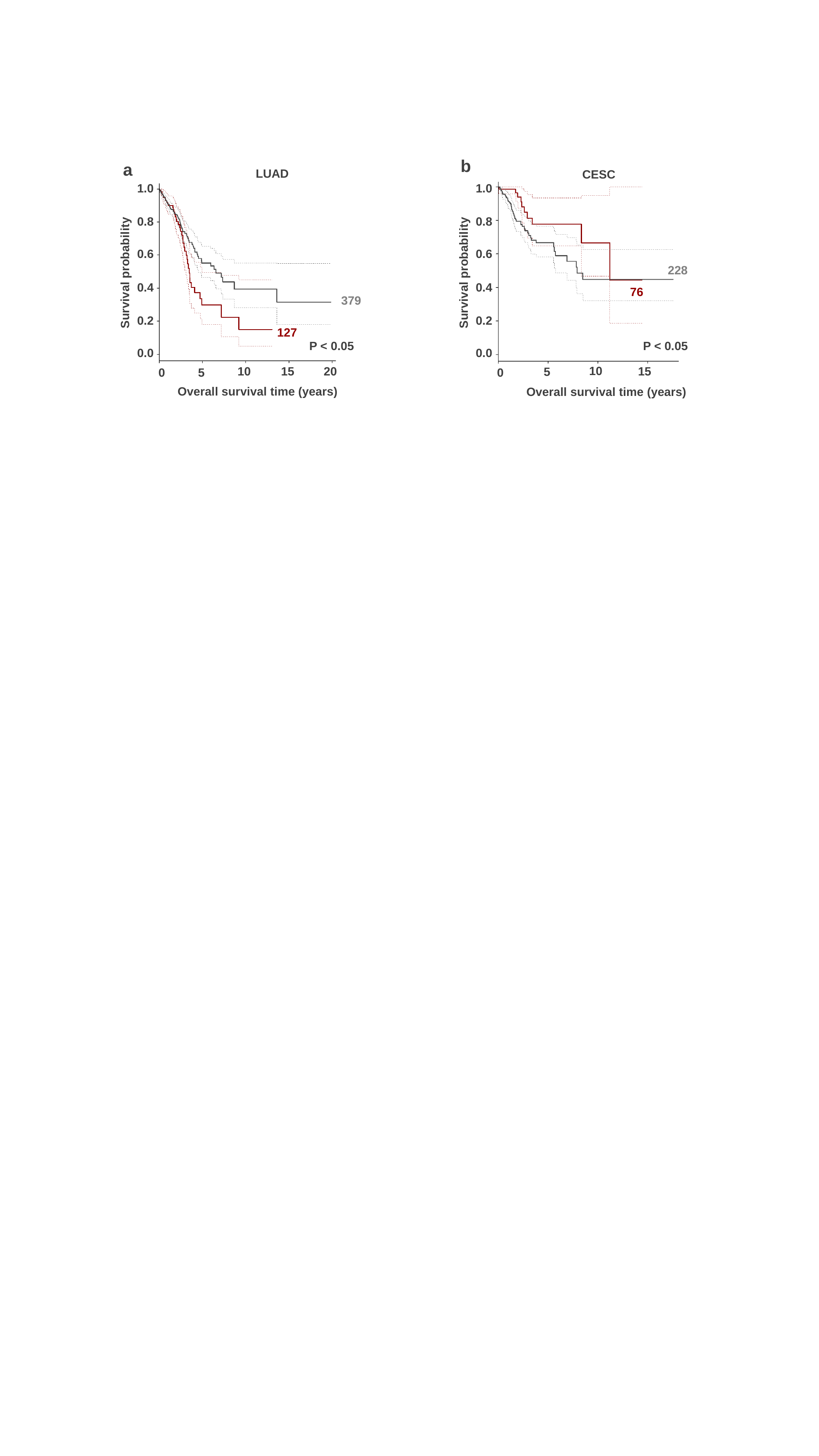

b
a
LUAD
CESC
1.0
1.0
0.8
0.8
0.6
0.6
228
Survival probability
Survival probability
0.4
0.4
76
379
0.2
0.2
127
P < 0.05
P < 0.05
0.0
0.0
10
15
10
15
20
5
0
0
5
Overall survival time (years)
Overall survival time (years)
